# Kinetics of Alkoxysilanes and Organoalkoxysilanes Polymerization: A Review

**DOI:** 10.3390/polym11030537

**Published:** 2019-03-21

**Authors:** Ahmed A. Issa, Adriaan S. Luyt

**Affiliations:** 1 Department of Chemistry and Earth Sciences, CAS, Qatar University, 2713 Doha, Qatar; ahmedissa@qu.edu.qa; 2 Center for Advanced Materials, Qatar University, 2713 Doha, Qatar

**Keywords:** silanes, polymerization, kinetics, characterization techniques, computational chemistry

## Abstract

Scientists from various different fields use organo-trialkoxysilanes and tetraalkoxysilanes in a number of applications. The silica-based materials are sometimes synthesized without a good understanding of the underlying reaction kinetics. This literature review attempts to be a comprehensive and more technical article in which the kinetics of alkoxysilanes polymerization are discussed. The kinetics of polymerization are controlled by primary factors, such as catalysts, water/silane ratio, pH, and organo-functional groups, while secondary factors, such as temperature, solvent, ionic strength, leaving group, and silane concentration, also have an influence on the reaction rates. Experiments to find correlations between these factors and reaction rates are restricted to certain conditions and most of them disregard the properties of the solvent. In this review, polymerization kinetics are discussed in the first two sections, with the first section covering early stage reactions when the reaction medium is homogenous, and the second section covering when phase separation occurs and the reaction medium becomes heterogeneous. Nuclear magnetic resonance (NMR) spectroscopy and other techniques are discussed in the third section. The last section summarizes the study of reaction mechanisms by using ab initio and Density Functional Theory (DFT) methods alone, and in combination with molecular dynamics (MD) or Monte Carlo (MC) methods.

## 1. Introduction

Silicone polymers (polysiloxanes) and other siloxane products are fabricated from alkoxysilanes (AS) and organoalkoxysilanes (OAS) through polymerization, which consists of poly-hydrolysis, re-esterification, and poly-condensation reactions. The final products and their applications depend significantly on the kinetics of these processes; a thorough understanding of the kinetics can therefore lead to control of the properties of the final product. The polymerization of alkoxysilane is a complex reaction. It consists of the following three major steps: (1) Hydrolysis, in which the hydroxyl group (OH) substitutes the alkoxyl groups in an acidic, alkaline, or neutral medium in the presence of a catalyst, using homogenizing agents (solvents); (2) condensation, which is the formation of siloxane bridges (Si-O-Si) by removing a water or alcohol molecule from two alkoxysilane molecules. Its kinetics are more complicated than that of hydrolysis, and it has many individual reaction rates. Condensation can occur between molecules and molecules, molecules and particles, or particles and particles; (3) phase separation, where the reaction medium loses its homogeneity to form a liquid in a continuous solid, or a solid (e.g., particles or precipitate) in a continuous liquid, which are, respectively, defined as a gel and a sol. It can also form silsesquioxanes (RSiO_1.5_)*_n_*. The kinetics of all these steps depend mainly on the water/Si ratio (R), catalyst, and nature of the silane, as well as the temperature, pH, solvent, ionic strength (IS), and silane/solvent ratio. Silane oligomerization kinetics are followed through many techniques, such as spectroscopy (nuclear magnetic resonance (NMR), FTIR, Raman, XRD, mass spectrometry (MS), UV/Vis, and X-ray photoelectron spectroscopy (XPS)), chromatography (liquid chromatography (LC), gas permeation chromatography (GPC), and gas chromatography (GC)), and microscopy (scanning electron microscopy (SEM), transmission electron microscopy (TEM), and atomic force microscopy (AFM)).

Large numbers of conditions can be used in sol-gel procedures, and these lead to variation in the properties of the produced materials due to differences in the kinetic parameters. Modelling and computational chemistry are therefore used to study the kinetics of certain silane precursors to help in understanding the kinetics. This is used to build a model, which can be used to theoretically predict the suitable conditions for fabrication of a certain material [1,2,3].

The kinetic parameters, polymerization stages, characterization techniques, and theoretical studies are discussed in more detail in this review, and we used the term, silane (SiH_4_), as a simplified reference to organoalkoxy- and alkoxy silanes.

For a more general understanding of the sol-gel process, and a more specific understanding of the polymerization of alkoxysilanes, there are a number of texts and literature reviews, which cover developments in the sol-gel technique up to 1997 [4,5,6,7,8,9,10,11,12,13,14,15]. Although this review covers developments after 1997, we shall periodically refer to these works because of their importance in understanding the basics of the technique. 

## 2. Notation

**Figure 1:**
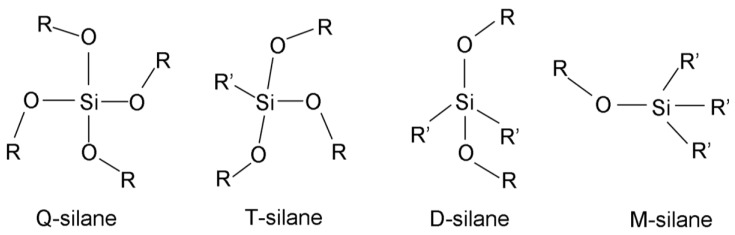
Scheme showing the different types of reacted silanes, where R is an alkyl and R’ is any organic group.

In this review, the letters, Q, T, D, and M, will be used to indicate the number of attached oxygen atoms on each silicon atom, where Q, T, D, and M, respectively, refer to four, three, two, and one oxygen atoms, regardless of the attached organic groups (Figure 1). They are written with numbers in different ways to express the number of hydrolyzed (Si-OH), un-hydrolyzed (Si-OR), and siloxane (Si-O-Si) bonds around the central silicon atom. The Q species will be used as an example to explain each type of notation. The simplest form that describes the number of hydrolyzed bonds in a silane monomer is Q*^x^*, where x is the number of Si-OH bonds and ranges from 0 to 4 [4]. Nowadays, this notation is used to refer to the number of siloxane bonds around the silicon atoms [16].

Q_0_(*x*,*y*) or Q^0^(*x*,*y*), the subscript or superscript, 0, indicating that it is a monomer, where *x* is the number of Si-OH, *y* is the number of Si-OR, and the sum of *x* and *y* is four. Q[*xyz*] is used to describe the central silicon atom in any form, where *x*, *y*, and *z* are the number of Si-OR, Si-OH, and Si-O-Si bonds, respectively. The sum of *x*, *y*, and *z* is also four. $Q_{x}^{n}$ is used to describe the produced monomer, dimer, trimer, etc. *n* is the number of Si-O-Si bonds surrounding each silicon atom, and x is the number of silicon atoms for each n species, e.g., $Q_{3}^{2}$ means a cyclic trimer. The last notation is used intensively in current publications, especially those that discuss silsesquioxanes. The most common abbreviations for the most used silanes, together with their chemical structures and properties, are listed in Table S1.

## 3. Hydrolysis and Early Stage Condensation

The polymerization reaction of silanes starts with hydrolysis of the silanes, followed by condensation to form a sol, a gel, or silsesquioxanes. The hydrolysis and condensation reactions are accompanied by re-esterification and de-polymerization reactions, respectively. All these reactions normally occur concurrently and consecutively, and each has its own kinetic parameters. It is well known that the polymerization of alkoxysilanes is a proton-transfer reaction [1,17,18], and it therefore undergoes an acid-base catalytic reaction. Hydrolysis kinetics have been extensively investigated, especially the first hydrolysis step, where the consumption of alkoxysilanes is normally easy to monitor, since it is assumed to be an elementary reaction. The first hydrolysis step also controls the overall polymerization, because it is the slowest step [19]. In general, the organo-alkoxysilanes’ polymerization can be represented by Equation (1):

(1) $$R_{x}^{\prime }\;\mathrm{Si}\;{\left(\mathrm{OR}\right)}_{4-x}+\left(4-x\right)H_{2}O\overset{\mathrm{hydrolysis}}{\to }R_{x}^{\prime }-\mathrm{Si}{\left(\mathrm{OH}\right)}_{4-x}+\left(4-x\right)\mathrm{ROH}\overset{\mathrm{condensation}}{\to }R_{x}^{\prime }{\mathrm{SiO}}_{\left(4-x\right)/2}+\left(4-x\right)/2H_{2}O$$

where *x* is an integer that ranges between 0 and 3. Polymerization is a composite reaction, and the reaction order cannot be related to the molecularity of the reaction. The molecularity of the reaction depends on the number of hydrolyzed groups (4 − *x*). The polymerization reaction steps of alkoxysilanes and organoalkoxysilanes are shown in Equations (2) to (5), in addition to the phase separation step.

1. Hydrolysis and re-esterification:
  ≡Si-O-R + H_2_O → ≡Si-OH + HO-R (2)
  ≡Si-OH + HO-R’ → ≡Si-OR’ + H_2_O (3)
2. Condensation by producing water:
  ≡Si-OH + HO-Si≡ → ≡Si-O-Si≡ + H_2_O (4)
3. Condensation by producing alcohol:
  ≡Si-OH + HO-Si≡ → ≡Si-O-Si≡ + HO-R(5)

As mentioned before, each of these steps (hydrolysis and condensation) has its own kinetic parameters, and each step has indefinite numbers of reactions close to or at equilibrium, due to polymerization and de-polymerization processes. The reaction rate of the first hydrolysis step can be expressed by Equation (6), while the observed rate constant (*k*_obs_) can be written as in Equation (7) [20]:

(6) $$rate=k_{\mathrm{obs}}\;{\left[\mathrm{alkoxysilane}\right]}^{n}{\left[\mathrm{water}\right]}^{m}$$

(7) $$k_{\mathrm{obs}}=k_{0}+k_{H}{\left[H^{+}\right]}^{i}+k_{\mathrm{OH}}{\left[{\mathrm{OH}}^{-}\right]}^{j}+k_{B}{\left[B\right]}^{k}+k_{\mathrm{HB}}{\left[\mathrm{HB}\right]}^{l}$$

where *k*_obs,_ *k*_0_, *k*_H_, *k*_OH_, *k*_B_, and *k*_HB_ are the observed, spontaneous, specific acid, specific base, general base, and general acid rate constants. *i*, *j*, *k*, *l*, *m*, and *n* are the reaction orders with respect to the proton, hydroxide, general base, general acid, water, and alkoxysilanes, respectively. In the following sections, we shall discuss the orders, mechanisms, and rates of the reactions in acidic, neutral, and alkaline media, as well as other parameters that will affect the polymerization reaction (e.g., catalysts, pH, molar ratio of water/silane). 

### 3.1. Reaction Order 

As will be discussed in the ‘catalyst’ subsection, the non-catalytic reaction is generally slow. The hydrolysis reaction is therefore a catalytic reaction, which depends on the concentrations of alkoxysilane, water, proton, general base/acid, and the produced alcohol. Most researchers avoid mentioning the reaction order, and they report the reaction rates qualitatively [21,22,23,24]. A number of researchers reported that the hydrolysis of silanes is first or pseudo-first order with respect to the alkoxysilane [25,26,27,28,29], while the order of the hydrolysis reaction with respect to water varied from 0.8 to 4.4 according to the solvent and catalyst used [4,29,30,31]. The condensation reaction was found to be second order in organosilanetriol, while the order of silicic acid condensation was found to be, respectively, third and fourth order in silicic acid concentration under biological and geological conditions [32,33]. Icopini [33] stated in the introduction of his paper that the order of condensation of silicic acid can range between 1 and 5, but it was found that the reaction was first order in the presence of a catalyst [19]. 

### 3.2. Mechanisms

**Figure 2:**
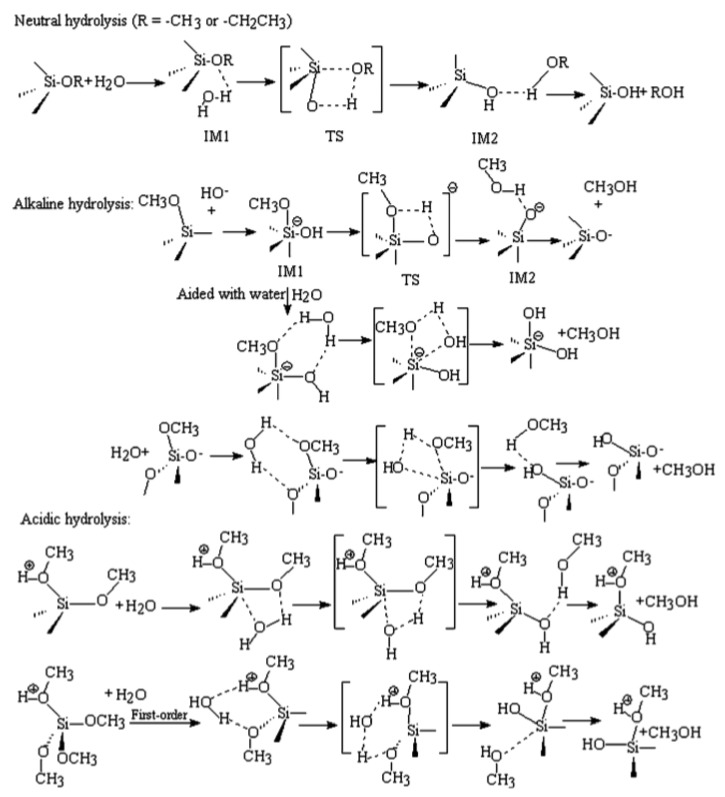
A schematic drawing of the hydrolysis mechanisms in different media. TS = transition state, IM = intermediate. Reprinted with permission from [35]. Copyright John Wiley and Sons, 2012.

The mechanisms of hydrolysis or condensation for all kinds of alkoxysilanes and organoalkoxy silanes are similar in the same reaction medium. However, they are different when the pH values of the media are different (Figure 2). In an alkaline medium, the nucleophilic hydroxyl or deprotonated silanol groups attack the silicon atoms in alkoxysilane and/or neutral silanol in hydrolysis and condensation reactions, respectively. The proposed reaction mechanism is S_N_2-Si with penta- or hexavalent intermediates/transition states. Steric and inductive factors affect the polymerization rates. The steric effect is more important in a condensation reaction, while the hydrolysis is quite sensitive to both the steric and inductive factors [4]. Note: There is an error in Figure 2, where the third line indicates the basic hydrolysis with additional water, with the product being a pentacoordinated anion, which is too reactive to be the product.

In an acidic medium, the alkoxide and silanol are first protonated in a fast step. The silicon atom then becomes more electrophilic and susceptible to backside attack by water or neutral silanol in, respectively, hydrolysis and condensation reactions. The reaction rate is affected by steric and inductive factors, as in the case of a base-catalyzed reaction. It was reported that the protonated silanol preferentially condensates with the least acidic silanol end groups, which leads to less branched clusters in an acidic medium. The deprotonated silanol, on the other hand, attacks the more acidic silanol groups, which leads to branched and condensed clusters in an alkaline medium [34].

Almost all the papers assumed an S_N_2-Si mechanism for silanes’ polymerization. Cheng et al. [35] confirmed through computational chemistry that the S_N_1-Si reaction mechanism is more favorable than the S_N_2-Si reaction mechanism. We think that the silanes undergo a polymerization reaction through an S_N_2-Si mechanism in alkaline media, and through an S_N_1-Si mechanism in neutral and acidic media. 

### 3.3. Reaction Rates

**Figure 3:**
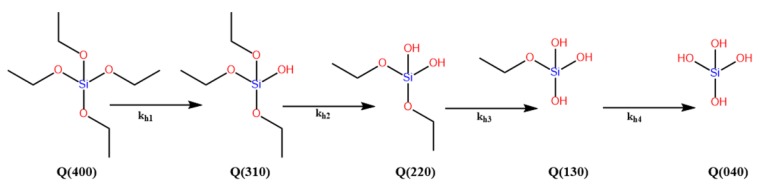
The hydrolysis of TEOS.

The polymerization rates of the alkoxysilanes can be expressed in different ways, depending on which reaction is monitored, e.g., first hydrolysis, overall hydrolysis, condensation, re-esterification, or gelation. The hydrolysis reaction can have up to four reaction rate coefficients (*k*_h_), depending on the existence of hydrolysable groups in the alkoxysilanes or organoalkoxysilanes, e.g., triethoxysilane (TEOS) (Figure 3). The rate constants could be in the ratio (4:3:2:1) [6], or they could have completely different values depending on the catalyst and solvent, as in the case of γ-glycidoxypropyltrimethoxysilane (GPS) [26]. Some articles, however, reported a single overall rate constant, *k*_h_, for the hydrolysis process [36,37].

The condensation reaction can have different rate constants, because the condensation has several condensation pathways, depending on the nature of the silanes and the reaction conditions. The rate constants can be expressed as $k_{\mathrm{cw}}$ and $k_{\mathrm{ca}}$, respectively, related to water or alcohol producing condensations [38], or $k_{c}$ for the overall condensation reaction rate constant [36]. The rate constants $\left(k_{h}\;\mathrm{or}\;k_{c}\right)$ vary largely depending on the reaction conditions and the types of silane. This will be discussed in detail in the following sections; some of the rate constants are listed in Table 1.

Rate constants were reported for hydrolysis, condensation, or both. Initially, some researchers used the Hammett and Taft equations to find a correlation between the hydrolysis rate and the alkoxysilane structures [31]. The hydrolysis rate was found to be enhanced as the pKa of the leaving group parent increased, while no correlation could be found between the organic appendages and the rate constants [39]. Recently, researchers tried to find empirical correlations between the rate constants by regression. The hydrolysis and condensation rates of TEOS in an alkaline medium were calculated by using Equations (8) and (9) [36]:

(8) kh=74.36exp(EaRT)[H2O]1.267[NH3]0.971

(9) kc=19408exp(EaRT)[H2O]1.196[NH3]0.7854

where, $k_{h}$ and $k_{c}$ are the hydrolysis and condensation rate constants, respectively. *E_a_* is the activation energy, which is equal to 25.2 and 33.2 kJ mol^−1^ in the case of hydrolysis and condensation, respectively. *R* is the universal gas constant, *T* the absolute temperature, and [H_2_O] and [NH_3_] are the water and ammonia concentrations, respectively. 

Analysis of the hydrolysis of TEOS in an acidic aqueous medium by using ultrasound gave *k*_h_ values of 4.6 × [H^+^]^−1^ M^−1^ min^−1^ at 39 °C [40] and 6.1 × [H^+^]^−1^ M^−1^ min^−1^ at 39 °C [41]. At a different ultrasonic power, the value was 0.276 × [HCl]^−1^ M^−1^ min^−1^ at 39 °C [30]. Aelion et al. [40] reported the hydrolysis rate constant for TEOS in dioxane as *k*_h_ = 3.06 × [HCl]^−1^ M^−1^ min^−1^ at 20 °C, where [HCl] and [H^+^] are, respectively, the HCl and proton concentrations.

### 3.4. Parameters that Affect the Hydrolysis-Condensation Kinetics

The polymerization of silanes can produce a variety of polymerized products by controlling the reaction conditions (parameters). Some of these parameters lead to an increase or decrease in the reaction rates, depending on the temperatures, while others, like changing the pH of the medium, completely change the kinetics of the reaction. It was found that the hydrolysis reaction was pseudo-first order with respect to alkoxysilanes, so the consumption of the alkoxysilanes at any time can be calculated by Equation (10):

(10) $$\left[\mathrm{alkoxysilane}\right]={\left[\mathrm{alkoxysilane}\right]}_{i}\;e^{-kt}$$

where [alkoxysilane] and [alkoxysilane]_I_ are the concentrations of alkoxysilane, respectively, at times (*t* and *t* = 0), while *k* is the rate constant. Equation (7) represents the rate constants in alkaline and acidic media, so it can be written as Equations (11) and (12) for acidic and alkaline media, respectively:

(11) $$k_{\mathrm{obs}}=k_{0}+k_{H}{\left[H^{+}\right]}^{i}$$

(12) $$k_{\mathrm{obs}}=k_{0}+k_{\mathrm{OH}}{\left[{\mathrm{OH}}^{-}\right]}^{j}+k_{B}{\left[B\right]}^{k}$$

During a spontaneous reaction, where there is no catalyst, the rate constant equals k_0_, and this depends on the solvent and the silane properties. With the addition of a catalyst, new parameters are added to the spontaneous condition parameters, which are related to the catalyst and its interaction with the solvent. These parameters will be discussed in the following sections.

#### 3.4.1. Catalysts 

In general, the polymerization of silanes is very slow without using catalysts [4], but there are exceptions, e.g., the use of 3-aminopropyltriethoxy silane (ATES) to make a film on the surface of copper. Here, it was shown that the amino group is involved in the sol-gel reaction as an intramolecular catalyst [49]. Different catalysts, such as ammonia, were used to improve the polymerization process to prepare monodisperse nanoparticles (Stöber method) [36,42,43,50,51,52]. Mineral acids, such as HCl [30,37,53,54,55,56,57,58], phosphoric acid [38], and hydrofluoric acid [59], as well as organic acids, such as acetic acid [2,23,26,45,60,61,62,63], chloroacetic acid [24], *p*-toluene sulphonic acid [22], and methane sulphonic acid [21], were also used. Researches also looked at the use of other organic compounds, such as tin alkyl esters [24,28], while boron-based catalysts were used to overcome the toxicity of the tin compounds. Other catalysts that were investigated were potassium carbonate [29], sodium hydroxide [64], and trimethyl amine (TEA) [65]. The use of ammonia and mineral acids as catalysts are well covered in the previous literature [4], and therefore we shall focus on the tin alkyl esters, boron-based compounds, sulphonic-based catalysts, and combined catalysts. 

Some sensitive equipment and structures require the hydrolysis of silanes in non-aqueous solutions in the absence of mineral acids. Torry et al. [26] used many organic and metalorganic catalysts to improve the hydrolysis of γ-glycidoxypropyl trimethoxy silane (GPS) in ethanol with 4% water. They found that the GPS hydrolysis without catalysts was extremely slow. The hydrolysis improved slightly when using acetic acid and other metal acetylacetonates (Table 2). However, real improvement was observed when dibutyltin dilaurate (DBTDL) and dibutyltin diacetate (DBTDA) were used. The hydrolysis rates with using alkyl tin esters approached the rates of using mineral acids.

**Figure 5:**
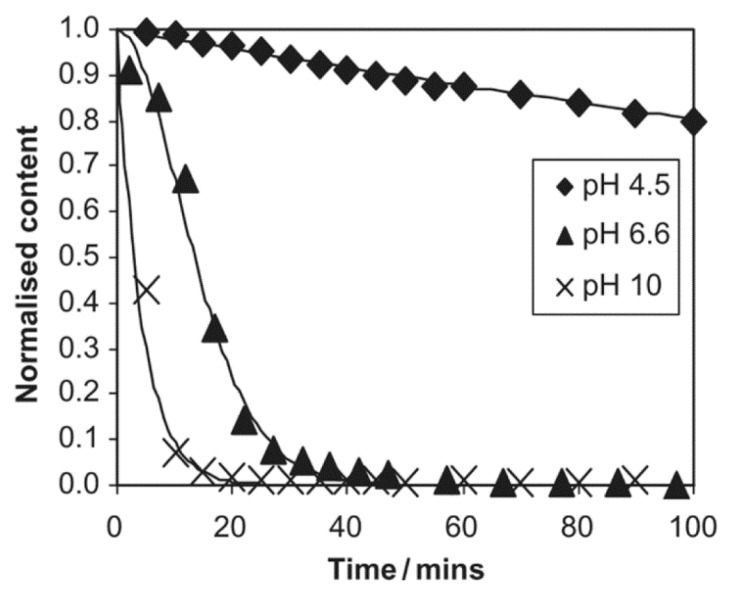
Consumption of GPS in different pH. Reprinted with permission from [26]. Copyright Elsevier, 2006.

DBTDL is an inactive catalyst, and requires an induction time before activation, while DBTDA is active and does not need an inductive period. Both of them are highly sensitive to the pH of the reaction medium [26] (Figure 5). Changing the catalyst not only affects the polymerization rate, but also the structural growth, as in the case of GPS. The authors reported that the cage structure fractions at a silicon to water ratio of 1.5 was 0.22, 0.45, or less than 0.05, depending on whether DBTDL, *p*-toluenesuphonic acid (TSA), or benzyl dimethyl amine (BDMA) was used as a catalyst [22].

The organotin compounds (especially DBTDL) were found to be excellent catalysts in non-aqueous systems [26,28]. However, researchers started looking for tin-free catalysts due to the toxicity of tin. They found that boron-based materials were promising catalysts [28]. Alkoxysilanes terminated polybutadiene was studied in the presence of different catalytic and co-catalytic systems from the following catalysts: diisobutoxy bis(ethylacetoacetato) titanate (Tyzor IBAY), 1,8-diazabicyclo[5.4.0]undec-7-ene(DBU), boron trifluoride monoethylamine (BF3-MEA) and tris(pentafluorophenyl)borane (BCF). A highly efficient co-catalytic system (BCF + TYZOR IBAY) was defined as a new tin-free catalyst for crosslinking applications [28]. Organic compounds (e.g. acetic acid) were used as catalysts, neglecting their solubilities and dissociation constants in organic aqueous mixtures, despite the importance of these parameters. 

#### 3.4.2. Water/Silane Ratio (r)

Stiochiometrically, the required water/silane ratio (r) to complete the polymerization of silanes depends on the number of hydrolyzable (alkoxide) groups in the silane molecule. The required ratio is 0.5, 1.0, 1.5, and 2.0 for M, D, T, and Q silane species, respectively. Increasing the water content improves the hydrolysis reaction up to a certain limit, after which further addition of water inhibits the reaction, probably because of the solubility of the alkoxysilanes [66]. The water content also determines whether the polymerization undergoes water or alcohol condensation. The structures of the produced solids further depend significantly on the water content in an acidic medium, while the water content does not have a noticeable effect in a neutral or basic medium [4,6]. The water is normally added to the system as liquid water, but it can also be introduced as humidity [28], steam [8], or through in situ water production from the esterification between chloroacetic acid and propanol [24].

**Figure 6:**
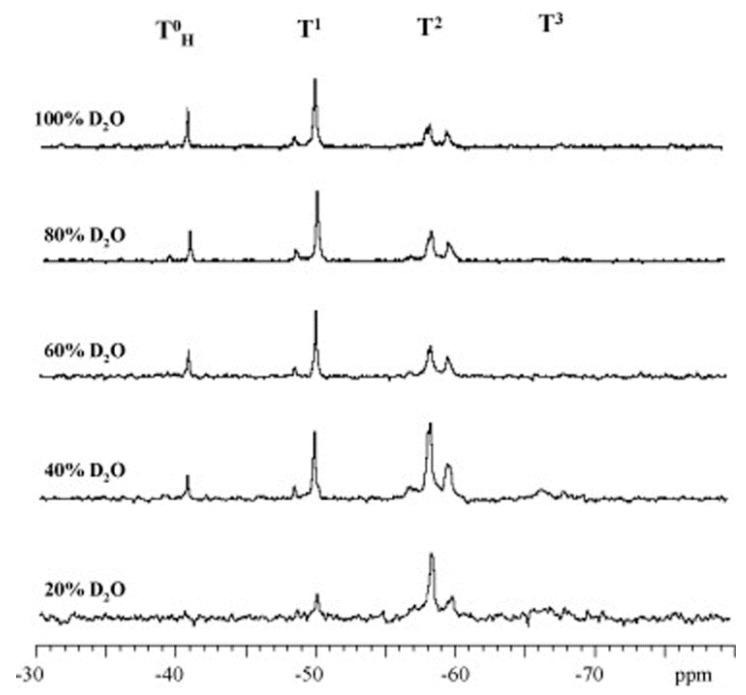
^29^Si NMR spectra of TAMS species in acidic media after 48 h. Reprinted with permission from [60]. Copyright Elsevier, 2010.

The water content was found to determine the percentages of the hydrolyzed species $\left(T_{H}^{0},\;T^{1},T^{2},\;T^{3}\right)$ in the reaction medium. An example is 3-[2-(2-aminoethylamino) ethylamino]propyl trimethoxy silane (TAMS), where the fully hydrolyzed monomer $\left(T_{H}^{0}\right)$ and dimer or chain ended group $\left(T^{1}\right)$ increased with the addition of water, while the highly condensed species $\left(T^{2}\;\mathrm{and}\;T^{3}\right)$ decreased with the addition of water (Figure 6) [60].

The water/silane ratio not only has an influence on the hydrolysis kinetics, but it also controls the oligomer structures, shapes, distribution, and molecular weights [8,22,29,56,58]. For example, the organotrialkoxysilanes have different percentages of linear/branched (A-series), monocyclic (B-series), and bicyclic (C-series) oligomers according to the water content. These oligomers have from 3 to 10 monomers [56]. It was reported that, with increasing the water content, the A-series content decreased, the B-series content rapidly increased, and the C-series content slowly increased (Table 3). Jai et al. [58] confirmed this to be true by observing that the A-series predominates at a silane/water ratio (r) of 0.6, while the B- and C-series predominate at r values of 1.0 and 1.2, respectively [58].

The molecular weight of the produced oligomers generally increased with increasing the water content [8,58]. 

#### 3.4.3. Chemical Properties of the Silane

The chemical properties of silane have a direct influence on the polymerization kinetics. Organoalkoxysilanes have the structure, $R_{x}\;\mathrm{Si}{\left(OR\prime \right)}_{\left(4-x\right)}$, where *x* is an integer with a value between zero and three. The organo-silanes are composed of organo groups (R) and leaving groups (OR’). According to Pohl et al. [19], the hydrolysis rate will increase if the hydrolysis reaction leads to a decrease in the crowding around the silicon atom, or to an improvement in the stabilization of the developed negative charge in the transition state. In general, as the leaving group tends to be larger and branched, the hydrolysis rate becomes slower, e.g., the hydrolysis rates of alkoxysilanes decreases in the order, mexthoxy > ethoxy > propoxy > butoxy silanes [4,67]. Altmann et al. [46] conducted a comprehensive study on the effect of the leaving groups on the hydrolysis rate of methacryl functional silanes in acidic and basic media. They used the silanes in Figure 6 and obtained the hydrolysis rates that are listed in Table 4. As expected, the methoxysilanes were more reactive than the ethoxysilanes in acidic and alkaline media (compounds **1a** and **2a** in Table 4).

The effect of the leaving group was also related to the thickness of the self-assembly monolayer (SAM) for the tested octadecyl silanes [68]. Identical conditions were used, except for changing the leaving group (methoxy, ethoxy, and chloro). It was found that the thickness of the SAM ranged between 0.8 to 2 nm according to the leaving group, which was attributed to the differences in the hydrolysis rate of the different silanes. 

Most of the conducted studies refer to the steric and inductive effects and polarity of the organo group (R), its reactivity, and the control of the polymerization rates depending on the pH of the reaction medium. The reaction rates were mainly governed by the competition between steric hindrance and inductive effects [4,46]. The presence of polarity was found to improve the solubility in the water by the formation of hydrogen bonds, enhancing the hydrolysis rate over the whole pH range. In some cases, the functional group in the organo group (R) was directly involved in the hydrolysis of the (Si-OR’) bond. 

The steric hindrance of the organo group generally slowed down the hydrolysis rates. Two research groups studied different kinds of amino trialkoxysilanes [47,60]. They found that the order of the hydrolysis rate for amine was primary > secondary > tertiary aminosilanes. They assumed that the crowding around the nitrogen atom in the secondary and tertiary amines decreased the hydrolysis rate. The slowing down of the silane hydrolysis by a steric effect is not restricted to the silanes, but it was also observed in the de-esterification of fatty acid esters [69].

**Figure 7:**
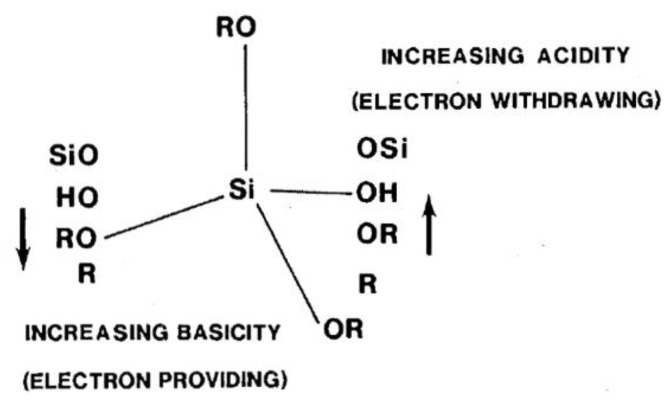
The inductive effect of different substituents on the hydrolysis in basic and acidic media. Repainted with permission from [4]. Copyright Elsevier, 1990.

It is well known that electron providing substituents increase hydrolysis in acidic media and decrease it in basic media, and vice versa [4,36,38] (Figure 7). This is known as the inductive effect. Many researchers reported that the organo group (R), which contained amino, carbonyl, or ester groups, enhanced the hydrolysis reaction [46,47,65] by interacting amongst themselves or with neighbouring silicon moieties. Salon et al. [23,60,62,65] studied the hydrolysis of different trimethoxy and triethoxy silanes at different conditions (pH, water content, etc.). Although they were not working quantitatively, they showed that the chemical structures had a direct impact on the hydrolysis rates and the distribution of the hydrolyzed species in the reaction medium. For example, they found that the hydrolysis rate was enhanced with increasing the number of amino groups in the silanes. They also reported that the transesterification (solvolysis) of some nitrogen bearing trialkoxysilanes with methanol and ethanol as solvents, in the absence of water and catalysts, depends on the chemical structure of the organo groups [23]. The hydrolysis rate of the (CH_3_)*_x_*–Si–(OC_2_H_5_)_(4 – *x*)_ silanes increased with increasing the substituent, *x*, which ranged from 0 to 2 in an acidic medium, which could have been due to an inductive effect [37]. Nearly the same results were obtained by Altman and co-workers [46]. 

#### 3.4.4. Temperature 

**Figure 8:**
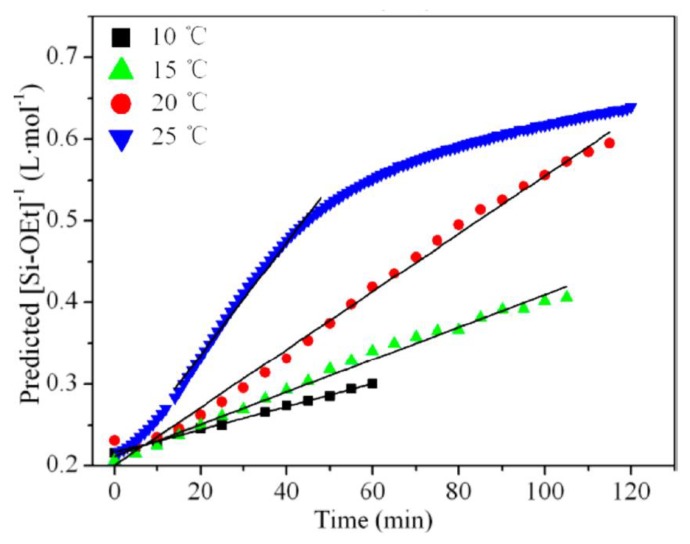
[Si-O-ethyl]^−1^ of MTES hydrolysis at different temperatures at [H_3_O^+^] = 1.468 × 10^−4^ M. Reprinted with permission from [37]. Copyright American Chemical Society, 2014.

It has generally been reported that hydrolysis, condensation, and cyclization reactions are endothermic under different conditions [2,17,70,71,72,73], while solvation and hydrogen bonding are slightly exothermic [17]. The polymerization reaction of silanes obeys the Arrhenius equation ($k_{h}=Ae^{\frac{-E_{a}}{RT}}$), where k_h_ is the rate constant, *E_a_* is the activation energy, *R* is the gas constant, and *T* is the absolute temperature. It is therefore expected that the polymerization of silane will be improved by using higher temperatures. Most researchers, who used heating during the reaction, noticed that the hydrolysis and condensation rates increased [37,38], e.g., methyltriethoxysilane (MTES) (Figure 8). Côrtes et al. [30] used ultrasound to enhance the hydrolysis of TEOS in water in the absence of a homogenizing agent and a catalyst. They found that the hydrolysis rate decreased with increasing the ultrasound power, which is contrary to what is expected. They referred this to bad coupling between the reactant phases with increasing the ultrasound power [30].

#### 3.4.5. Solvents

Solvents are used as homogenizing agents in the polymerization of silanes. Three parameters determine the power of the solvents: Polarity, dipole moment, and availability of labile protons. The solvents are divided into polar and non-polar, and into protic and aprotic. The polarity affects the solubility of the silanes in the reaction mixture. The protic and aprotic properties determine the stability of the hydroxyl and hydronium ions in the reaction medium, depending on the pH, and aprotic solvents are assumed to be inert solvents. Protic solvents improve the condensation in acidic media, but decrease it in basic media; the reverse effect was noticed for aprotic solvents. Finally, increasing the solvent viscosity reduced the hydrolysis rate due to decreasing the diffusion coefficient of the reactants [4]. Ethanol is a common solvent in the sol-gel method, especially in the Stöber method (preparation of silica particles in the presence of concentrated ammonia). Several other solvents, like dimethyl formamide (DMF), carbon tetrachloride, tetrahydrofuran (THF), alcohol, and acetone, were also used.

**Figure 9:**
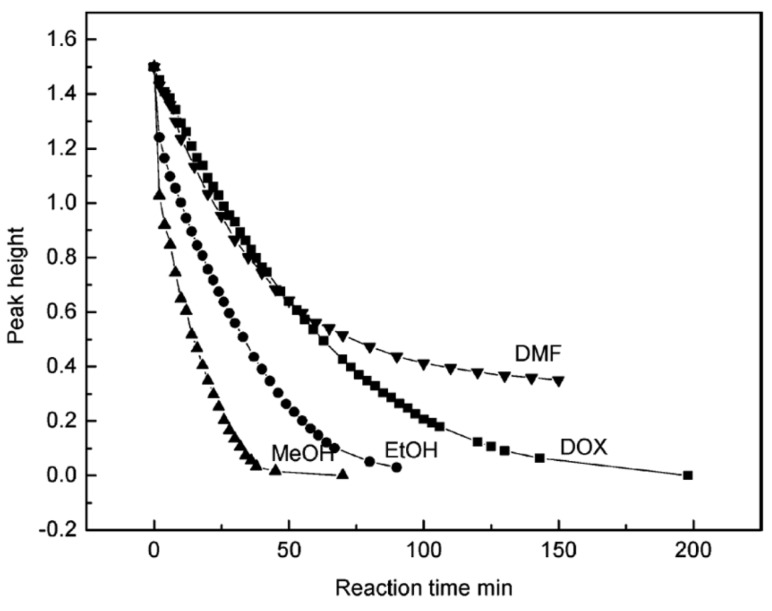
Kinetics of hydrolysis for MTES in an alkaline system for different solvents, inferred from the peak height of MTES at 960.0 cm^−1^. Reprinted with permission from [48]. Copyright American Chemical Society, 2006.

The effect of different solvents (methanol, ethanol, DMF, and dioxane) on the hydrolysis rate of methyl trimethoxy silane (MTMS) was studied in an alkaline medium [48]. It was reported that the times for the complete disappearance of MTMS were 30, 60, 120, and >150 min in methanol, ethanol, dioxane, and DMF, respectively (Figure 9). Similar results were obtained for the hydrolysis of TEOS in methanol and ethanol under alkaline conditions [42]. The addition of methanol to a methanol/water solvent mixture significantly decreased the hydrolysis rate of GPS, as well as the produced amounts of oligomers [45]. 

It was mentioned in [4] that protic solvents make the hydronium more electrophilic and the reaction medium becomes more acidic with the addition of alcohol, so that the hydrolysis rate should be enhanced by increasing the alcohol concentration. Abel et al. [45], however, showed that the hydrolysis rate of GPS decreases with increasing the methanol content in the water. Subcritical and supercritical carbon dioxide was also used as solvents to coat titanium dioxide nanoparticles in order to avoid the production of low-quality SAM when regular solvents were used [44]. The grafting rate under supercritical conditions was found to be very fast and to lead to the formation of 2D layers. Grafting in subcritical CO_2_ was slower and was controlled by the concentration of silane and the availability of hydroxyl groups on the surfaces of the titanium nanoparticles. The grafting percentage could be tuned by varying the time under subcritical conditions. 

#### 3.4.6. Ionic Strength 

In kinetics studies, salt addition has a primary and secondary effect. In the primary effect, the salt influences the activity coefficients of the reactant and the activated complex, while in the secondary effect, the salt becomes a part of the equilibrium equations, especially in acid-base catalyzed reactions, such as alkoxysilanes polymerization. Usually, the addition of salt is investigated as a catalyst, as was shown in the catalyst section. Only Pohl et al. [19] studied hydrolysis under controlled ionic strength during the early stages of the condensation of organoalkoxysilanes, in order to eliminate the secondary effect of salts during the investigation of the proton effect. However, geologists and some biologists have carefully studied the formation of silica in nature [33,74,75,76]. They found that the ionic strength had a direct influence on the stability of nano-colloidal silica, which will be discussed in another section. Unpublished work indicated that salts had a complex effect. It behaved as an acid or base, depending on whether it contained a strong conjugated acid or base, and a weak acid or base, e.g., ammonium chloride (sodium acetate). It also changed the reaction order in silane with the addition of neutral salts (e.g., sodium chloride).

#### 3.4.7. pH

**Figure 10:**
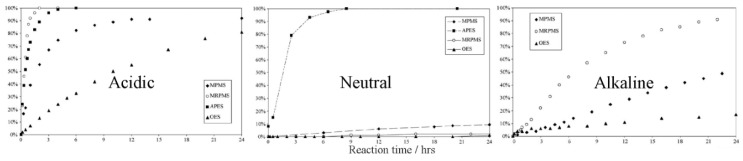
Comparison of hydrolysis rates of MPMS, MRPMS, APES, and OES in different media. Reprinted with permission from [65]. Copyright Elsevier, 2008.

Polymerization kinetics depend on the pH of the reaction medium. In an acidic medium, the hydrolysis is very fast and the condensation is very slow, while in a neutral medium, both of them are slow, except in the case of amino silanes. In an alkaline medium, the hydrolysis is very slow and the condensation is very fast. Therefore, either the hydrolysis or the condensation forms the limiting step, depending on the pH of the medium [40,65]. It was further shown that the hydrolysis rate of TEOS depends on the proton [H^+^] concentration, regardless of the solvents used or the use of sonication [41]. As was shown in Section 3.3, the hydrolysis reaction rate is proportional to the ammonia concentration [NH_3_], and inversely proportional to the proton [H^+^] concentration. Despite the fact that the hydrolysis rate for different silanes (e.g., dimethyl dimethoxysilane (DMDEOS), MTES, and TEOS) may decrease or increase according to the pH, the order of the hydrolysis rates are the same for a specific pH in an acidic medium [37]. However, the order of hydrolysis rates of γ-methacryloxypropyltrimethoxy (γ-MPS), 3-mercaptopropyl trimethoxy silane (MRPMS), octyl triethoxy silane (OES), and 3-aminopropyl triethoxy silane (APES) are completely different, depending on whether the medium is acidic, neutral, or alkaline (Figure 10) [65]. The aliphatic substituents have the lowest hydrolysis rate in all the media, although it is faster in an acidic medium. However, the hydrolysis rate of the amino substituent is better in a neutral than in an acidic medium, due to the consumption of the acid in the neutralization of the amino group.

#### 3.4.8. Silane Concentration 

**Figure 11:**
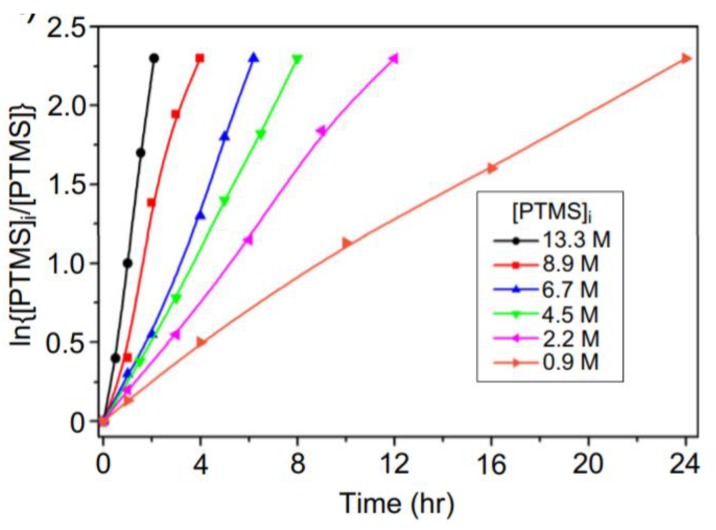
Kinetic rate plots for various [PTMS]*_i_*, [H_2_O] = 30 M, at 25 °C. Reprinted with permission from [29]. Copyright Elsevier, 2016.

As mentioned earlier, the hydrolysis reaction of silanes is first order or pseudo-first order [25,26,27,28,29,36,37,43,50]. Therefore, by increasing the initial concentration of the silane in PTMS, the reaction rate will increase (Figure 11) [29]. In addition, the molecular weight of the PTMS silsesquioxanes is increased as the concentration of PTMS increases up to a certain limit (13.3 M), after which it again decreases [29]. This is in line with the results reported in [77], where the hydrolysis and condensation of γ-MPS was found to depend on the pH and initial concentration of silanes, respectively.

This section summarized work on the polymerization reactions when the reaction medium was still homogenous. It discussed the factors that were found to affect the reaction rates, as well as some efforts to find an equation to predict the hydrolysis rate. However, most of these works were descriptive and focused on one parameter at a time. The effect of the IS received attention in studies on the phase separation kinetics, but it was neglected in studies on early stage reactions. There is, however, no report on efforts to find an equation which relates the reaction rate to several reaction conditions.

## 4. Phase Separation

It is known [4] that the polymerization of silanes starts with hydrolysis, followed directly by condensation. In the presence of excess water, the condensation during the early stages of the reaction forms dimers and trimers, which are soluble in the reaction medium. Further condensation reactions give rise to the dimers and trimers growing into oligomers, and then into macromolecules. The reaction medium is usually homogenous in the early stages of the polymerization, and starts to be heterogeneous as the condensation or aggregation proceeds. This leads to phase separation, which could be a colloidal suspension of particles in a liquid (sol), a continuous solid in a continuous liquid (gel), or immiscible oligomers in a liquid (silsesquioxanes). The phase separation depends on many parameters, especially the silane functionality, the pH, and the catalyst. Silane functionality is the ability of the silane monomer to form siloxane bonds (Si-O-Si), ranging from one to four. Mono-functional silane (M) can form a dimer, and the reaction medium will be homogenous, while bifunctional silanes (D) produce linear/cyclic polymers. Trifunctional silanes (T), however, produce crosslinked oligomers and polymers (silsesquioxanes). The tetra-functional silanes (Q) form highly crosslinked polymers that can be sol or gel depending on a number of parameters. A book on this topic [4] gives a detailed discussion of the sol and especially the gel. In the following sections, we shall discuss the sol, gel, and silsesquioxanes.

### 4.1. Sol (Stöber Particles)

The theoretical principles of sol formation of metal oxides in general and silica in particular were discussed in a previously published text [4]. They discuss kinetic growth models, in which they describe monomer-to-cluster addition and cluster-to-cluster addition in basic and acidic media. They also discuss the Ostwald ripening of the primary particles, and models for the formation of the Stöber particles. Under the conditions used in the Stöber method, the formation of monosized spherical particles is reaction-limited by monomer-cluster aggregation [4]. The particles are formed in two steps, with the small primary particles (<6 nm) forming through nucleation, growth and ripening, and then the final particles grow through aggregation. 

A supersaturated concentration of the monomers and solubility of the particles normally gives rise to dense primary particles. Nuclei are formed rapidly above a critical concentration. If the formed particles are too small, they are unstable and will re-dissolve and re-form until the critical diameter is exceeded. The solubility of the particles is expressed in Equation (13):

(13) $$S=S_{0}\exp\left(\frac{2\gamma_{\mathrm{LS}}\;V_{m}}{R_{g}Tr}\right)$$

where *S* is the solubility, $S_{0}$ the solubility of the flat particles, $\gamma_{\mathrm{LS}}$ the solid-to-liquid interfacial energy, $V_{m}$ the molar volume, *R*_g_ the gas constant, *T* the absolute temperature, and *r* the radius. There is aggregation between the formed particles, where the small particles are attracted to the big particles, even if they have the same charge, and attractive and repulsive forces control the stabilization of the particles in aqueous medium. The attraction is due to Van der Waals forces (Equation (14)):

(14) $$V_{a}=\left(-A/12\pi h^{2}\right)$$

where *A* is a material property called the Hamaker constant, and *h* is the distance between the two particles. The repulsive force, $F_{R}\propto ke^{-k\left(h-H\right)}$, is due to electrostatic repulsion in the double layer around the particle, where *h* is the thickness of the Stern layer, *H* the distance between the slip plane in the Gouy layer and the surface of the particles, and *k* can be calculated from Equation (15):

(15) $$k=\sqrt{\frac{F^{2}\;\sum_{i}C_{i}\;Z_{i}^{2}}{\varepsilon \varepsilon_{o}R_{g}T}}$$

where *F* is the Faraday constant, *C* and *Z* are the concentration and charge of the counter ions, ε is the dielectric constant of the solvent, and $\varepsilon_{o}$ is the vacuum permittivity. The formation of the particles is therefore controlled by many parameters.

Bogush et al. [43] studied the formation of silica particles, and they tried to test the hypothetical mechanisms of growing monodispersed spherical particles in the submicron range. The first mechanism was the LaMer and Dinegar mechanism, in which the particles were formed by a short burst of nucleation followed by self-sharpening. After the nucleation, the smaller particles grew faster than the bigger ones, while the mass transfer was controlled by diffusion of the reactant to the surface of the particles. Secondly, in the aggregation mechanism derived from the colloidal theory of growing latex particles, the freshly formed nuclei are colloidally unstable and grow with aggregation to a size at which the growth is stopped. Matsoukas and Gulari [78,79] hypothesize that the nuclei are formed from the condensation of two silicic acid (Si(OH)_4_) particles, after which the particles grow with monomer addition. In this case, the hydrolysis is the limiting reaction step. Their work is consistent with the LaMer and Dinegar model. The last theory is the Flory-Stockmayer theory, in which the silicic polymer will grow to a certain molecular weight, after which the growing polymer is unstable and phase separation occurs to form dense particles (nucleation), and these particles grow through aggregation. Bogush et al. [43] found that the mechanism that depended on monomer diffusion to the particle surface was not accurate. Their results did not support the LaMer and Dinegar mechanism, but were more consistent with the aggregation model. It is probably because the nuclei will not stay in the solution and grow by monomer diffusion, because particles with sizes less than 20 nm are not colloidally stable [4,43]. They finally suggested that, in a basic medium, the hydrolysis is the limiting reaction, while condensation proceeds for the primary particles (nuclei) following a Flory-Stockmayer pathway. Under certain circumstances, when the primary particles are colloidally stable, the particles’ growth follows the LaMer model, but otherwise the particles grow through aggregation [50]. They developed an equation to predict the size of the silica particles depending on the concentration of ammonia, water, and TEOS [51]. Razink et al. [52] added a small correction to the equation, so Equations (16) to (18) are now regarded as the final equations to calculate the average diameter, *d*:

(16) $$d=A{\left[H_{2}0\right]}^{2}\exp(-B{\left[H_{2}O\right]}^{0.5}$$

(17) $$A={\left[\mathrm{TEOS}\right]}^{0.5}\left(82+151\left[{\mathrm{NH}}_{3}\right]+1200{\left[{\mathrm{NH}}_{3}\right]}^{2}-366{\left[{\mathrm{NH}}_{3}\right]}^{3}\right)$$

(18) $$B=1.05+0.523\left[{\mathrm{NH}}_{3}\right]-0.128{\left[{\mathrm{NH}}_{3}\right]}^{2}$$

where [TEOS], [NH_3_], and [H_2_O] are the concentrations of the TEOS, ammonia, and water, respectively.

**Figure 12:**
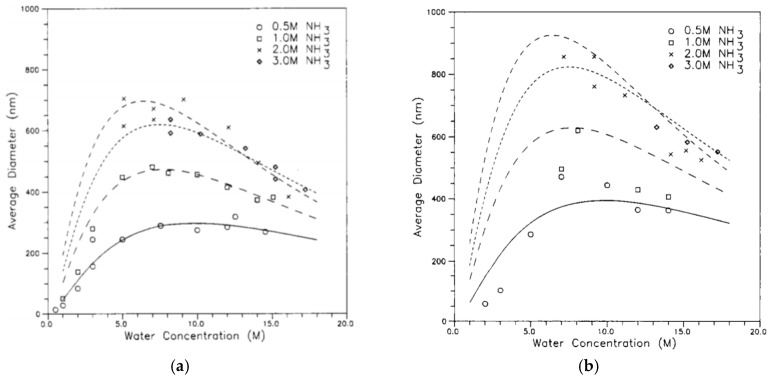
The relation between water content and average particle diameters at different ammonia concentrations at constant temperature and concentration of TEOS: (**a**) 0.17 M and (**b**) 0.3 M. Reprinted with permission from [51]. Copyright Elsevier, 1998.

Equations (16) to (18) show that the concentration of ammonia, water, and TEOS have a direct effect on the dimeter of the produced silica particles (Figure 12).

**Figure 13:**
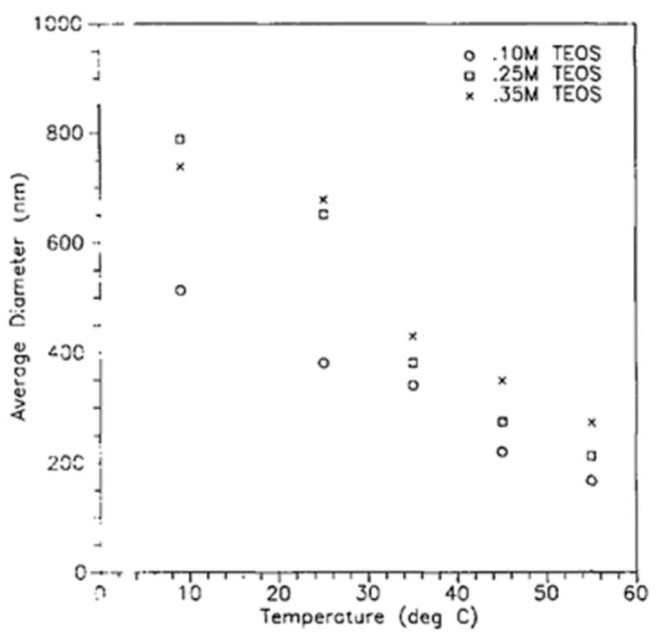
Relation between the temperature and particle size at different concentrations of TEOS. Reprinted with permission from [51]. Copyright Elsevier, 1998.

Increasing the temperature led to a reduction in the size of the produced particles (Figure 13). Increasing the ionic strength or changing the pH of the medium led to a collapse of the double layer, which decreased the repulsive force, and which could increase the particle sizes or could give rise to the formation of a gel [4,50,80].

**Figure 14:**
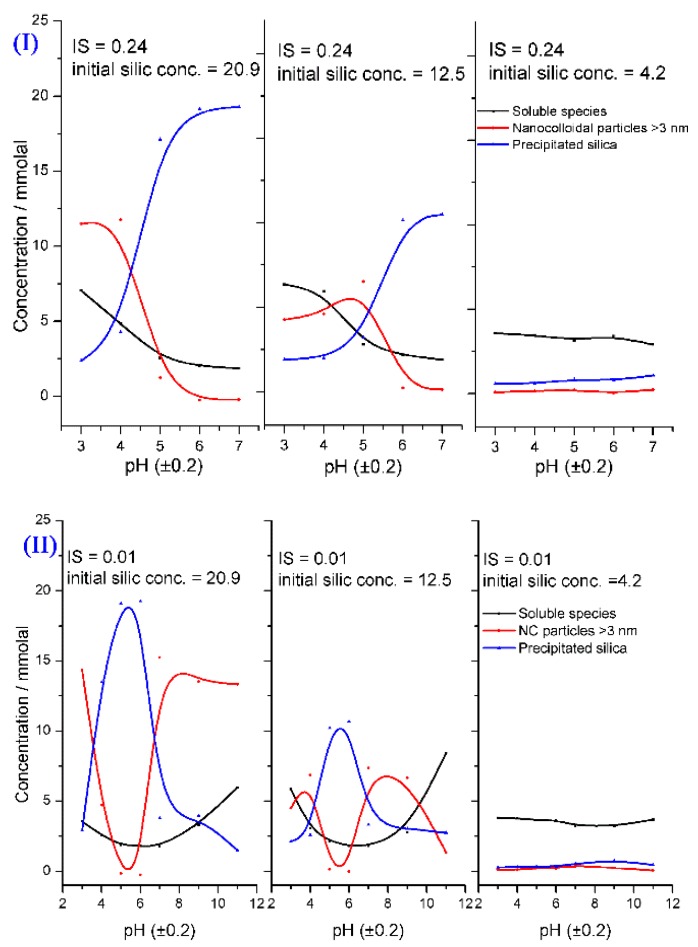
Extracted data represent the silica species as a function of the pH at different concentrations of (**I**) silicic acid and (**II**) IS. Reprinted with permission from [33]. Copyright Elsevier, 2005.

Icopini et al. [33] studied the effect of the ionic strength (IS), pH, and concentration of silicic acid on silica oligomerization at 25 °C. The silica species were soluble silica monomers (monomer, dimer, and trimer) ([SiO_2_]*_n_*_≤ 3_), nano-colloidal particles ([SiO_2_]*_n_*_> 3_), and precipitated silica ([SiO_2_]_ppt_). Their results can be summarized as follows:

a) The silica particles were colloidally stable between pH 2 and pH 3. They had a minimum stability at pH 7, and they became highly soluble at pH 11.
b) Increasing the IS decreased the required time to reach the equilibrium state, regardless of the pH and the initial silica concentration.
c) The concentrations of the silica species depended on the pH, IS, and initial concentration of the silicic acid (Figure 14).
d) The silicic acid polymerization rate increased near neutral and with increasing the IS. An equation describing the relation between rate constant, $k_{4}$, pH, and concentration (Equation 19) was proposed:
  (19) $$\log k_{4}=mpH+\log k_{0},\;\mathrm{and}\;k_{4}=\left(\frac{1}{3t}\right)(\frac{1}{C^{3}}-\frac{1}{C_{0}^{3}})$$
  where m is an empirical value which is positive at pH < 7 and negative at pH > 7, $k_{0}$ is the rate constant at pH equal to zero, *t* is the time, $C\;\mathrm{and}\;C_{0}$ are the concentrations of silicic acid at time *t* and initially, respectively. The addition of IS can affect the transfer of the sol to the gel, but at a certain IS concentration, the addition of the IS leads to the stabilization of the produced silica particles. It was found that silica particles with small sizes could be fabricated by using high concentrations of different chloride and potassium salts [81]. Moreover, the kinetics of the sol and gelation were affected by the Hofmeister series of counter ions [82,83,84].

### 4.2. Gel

**Figure 15:**
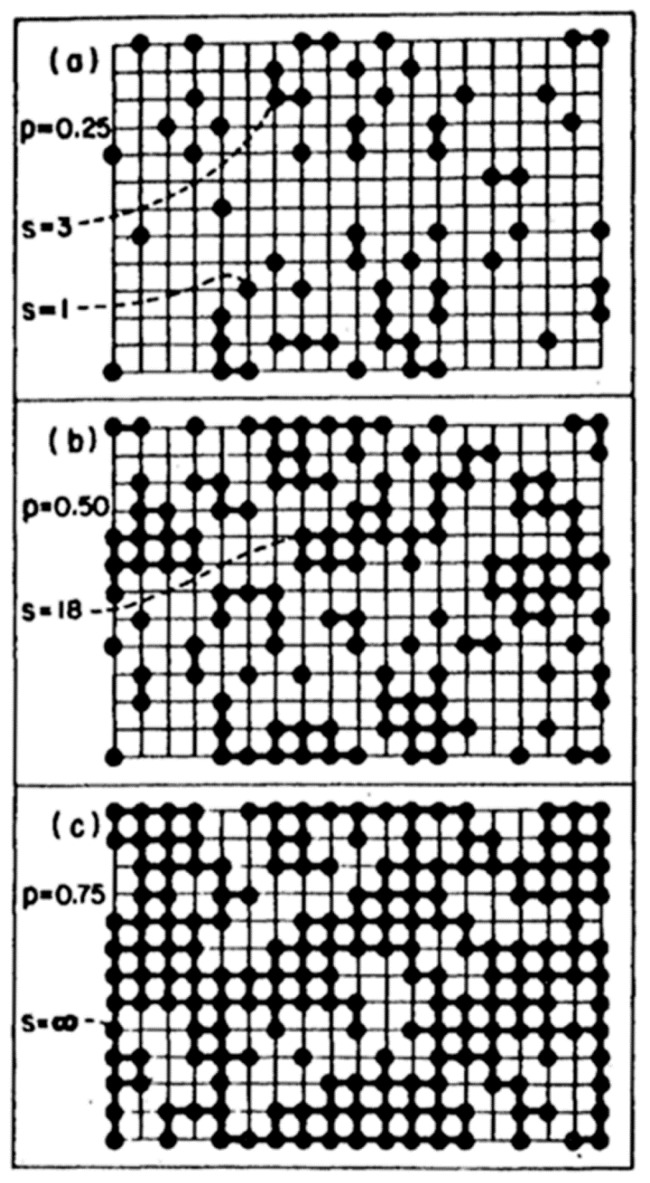
The schematic explanation of site-percolation in a 2D lattice, where the fraction of filled sites (p) are (**a**) 0.25, (**b**) 0.50, and (**c**) 0.75 (gelation point). Reprinted with permission from [4]. Copyright Elsevier, 1990.

As defined before, gel is a continuous solid in a continuous liquid produced from linking the small clusters in the solution to form a giant cluster, which connects the two sides of the container. In a special case, it is produced from aggregation of particles after collapsing the protective double layer around the particles due to changing the pH or increasing the IS of the solution. Two theories are used to explain the formation of the gel. The first is the classical theory (Flory and Stockmayer), where the polymer with tetra-functional groups grow as a tree. The following are assumed: (i) All the active sites have equal reactivity; (ii) the linking does not occur within the same polymer (no closed loop) [4]. Depla et al. [16] showed that the gelation is a competition between cyclization and chain extension, which depends greatly on the water concentration [16]. The second is the percolation theory, which was developed to avoid the unrealistic assumption of the classical theory about the absence of a closed loop inside the cluster. If the polymer grows as a tree in the classical theory, it grows on a gird in the percolation theory, and each intercept on this grid (lattice) represents a site for the monomer. When any two neighbour sites are filled, they will be connected. With the addition of monomers to the grid, they will continue connecting until a critical point (percolation threshold) is reached, at which a giant cluster will appear, and this is the gel point (Figure 15). The preceding description is called site percolation. There are also bond, site-bond, and correlated site-bond percolations. In site-bond and correlated site-bond percolations, the presence of the solvent molecules, the interaction energy between them, and the silane monomer are considered.

**Figure 16:**
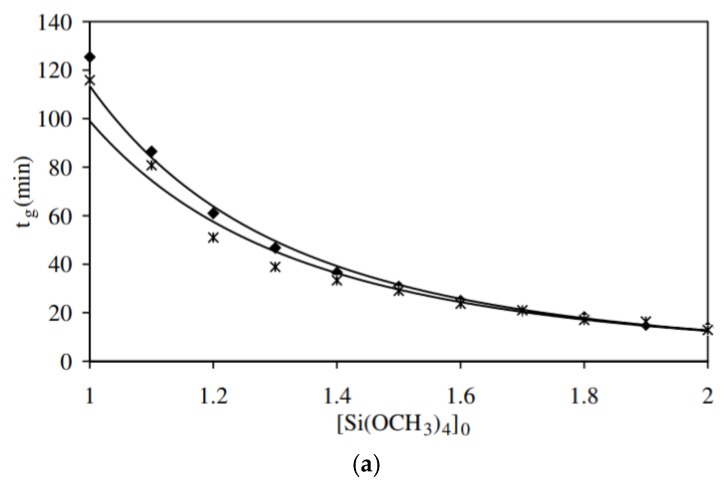

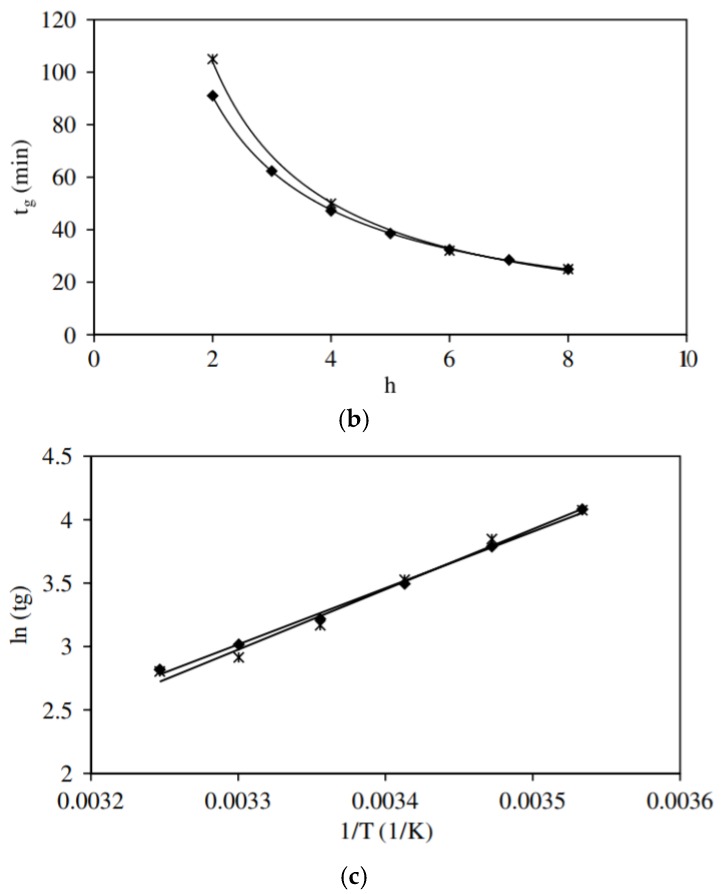
The gelation time (*t*_g_) of the TMOS at different (**a**) initial concentrations of TMOS, (**b**) water/silane ratios (*r* or *h*), and (**c**) temperatures. Reprinted with permission from [85]. Copyright Elsevier, 2003.

It was reported that gelation is a second order reaction [11], and that the degree of condensation *C*(*t*) has three different regions according to the reaction time. Below the gelation time (*t*_g_) it has a small variation due to the formation of dense polyhedral structures. Between *t*_g_ and *2t*_g_, the *C*(*t*) increases linearly with time. Between *2t*_g_ and *8t*_g_, which is the saturation region, *C*(*t*) was found to be equal to 0.89. The parameters that influence the gelation time are the water/silane molar ratio (r-value), initial concentration of silane, temperature, catalyst, addition of organo-silanes, and types and concentrations of salts. Generally, increasing the r-value, temperature, and initial concentration, decreases *t*_g_. Equations (20) to (22) [85], which describe the relation between the gelation time and these three parameters, were developed:

(20) $$t_{g}=C{\left[\mathrm{Si}{\left({\mathrm{OCH}}_{3}\right)}_{4}\right]}_{0}^{-\alpha }$$

(21) $$t_{g}=Bh^{-\beta }$$

(22) $$\ln\left(t_{g}\right)=A^{\prime }+\frac{E_{a}^{\prime }}{RT}$$

where, $\left[\mathrm{Si}{\left({\mathrm{OCH}}_{3}\right)}_{4}\right]$ is the concentration of tetramethoxy silane (TMOS), C = 113 $mM^{-\alpha }$, α = 3.16, and the correlation coefficient (*R*^2^) is 0.994. B = 173, β = 0.93, and *R*^2^ = 0.998. $A^{\prime }$ = 0.03 l/m, $E_{a}^{\prime }$ = 37 kJ mol^−1^, and *R*^2^ = 0.996 by using the rheology technique (Figure 16).

A few researchers reported that the addition of organotrialkoxysilanes to TEOS or TMOS leads to an increase in the gelation time due to steric effects [86,87]. For example, the addition of γ-MPS, phenyl triethoxysilane (PhTES), methyl trimethoxy (MTMS) [86], and phenyl aminotriethoxysilane (PAMS) [87] to TEOS increased the gelation time. 

There are two points on the pH scale at which the aggregation of the particles should be relatively high. The first is the isoelectric point (IEP), at which the electric potential on the surface is zero (no repulsive force), while the second is the zero charge point (ZCP), at which the particles are neutral (not positive or negative). However, this is not the case with silica particles, since they do not aggregate at the IEP due to the formation of a water layer around the particles. The used catalysts have a direct impact on the gelation time [4,76], and the combination of other parameters (solvent, catalyst, etc.) with the pH can control the properties of the produced silica [76].

The ionic strength is important in the gelation process, starting from its concentration to the chemical properties of the counter ions. IS usually destroys the protection of the repulsive double layer around the primary particles, resulting in particles’ aggregation and the formation of a gel. However, increasing the IS above 0.1 M leads to stabilization of the particles and prevention of the aggregation due to the conversion of the charge on the particle surface and the creation of its own repulsive double layer [4]. As mentioned in the preceding section, the Hofmeister series have an effect on the gelation kinetics [84]. The effect of three anions (cyanide, chloride, and iodate) of potassium salts on the gelation time in an acidic medium was studied, and it was found that the gelation times were in the order of KSCN < KCl < KIO_3_ under the same conditions and concentration. Another group obtained the same result, where they used potassium (K), lithium (Li), sodium (Na), cesium (Cs), and rubidium (Rb) chloride salts in a basic medium. The gel time was reported to be in the order of CsCl < RbCl < KCl < NaCl < LiCl [82].

### 4.3. Silsesquioxanes 

**Figure 17:**
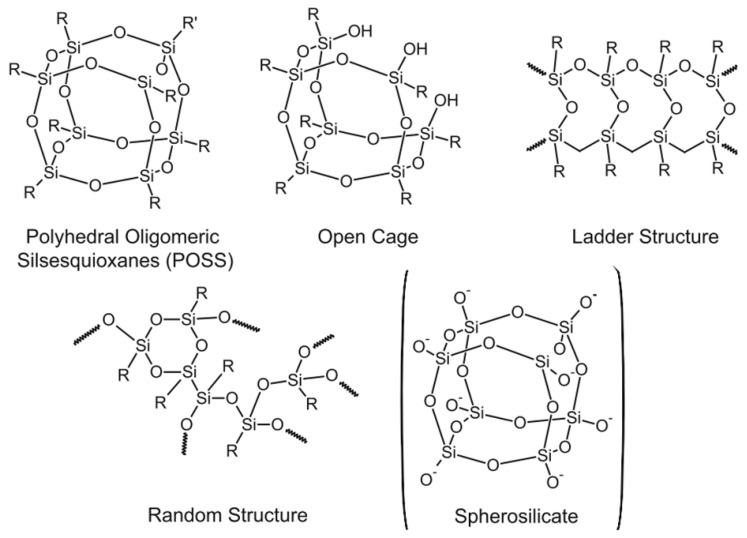
The structures of some silsesquioxanes. Reprinted with permission from [9]. Copyright Springer Nature, 2003.

The organotrialkoxysilanes are especially important in many applications, e.g., protective coatings on metal surfaces, adhesive prompters, and coupling agents. These silanes have an inorganic part (silicon) and an organic part (organic functional groups) that can lead to control of the interfaces between the metals and their surroundings [67]. Even the incompletely condensed silsesquioxanes are used as catalysts for the polymerization of olefins [14]. If the polymerization of tetra-functional silanes leads to silica particles or a gel, the condensation of the tri-functional silanes forms silsesquioxanes with different structures, e.g., polyhedral oligomeric silsesquioxanes (POSS), open cage, ladder, and random structures, with different molecular weights depending on the reaction conditions (Figure 17). Most of the produced silsesquioxanes are obtained from organotrichlorosilanes rather than from organotrialkoxysilanes [9,21,29,88,89]. Temperature is the critical parameter in the formation of silsesquioxanes, where a highly condensed polymer is formed at high temperatures, so most silsesquioxanes are fabricated at low temperatures close to 0 °C [9]. Solvents and catalysts have an effect on the structure of the produced silsesquioxanes. The formation of silsesquioxanes was discussed fairly extensively in previously published texts [67]. In this work, we will focus on the silsesquioxanes produced from organotrialkoxysilanes.

**Figure 18:**
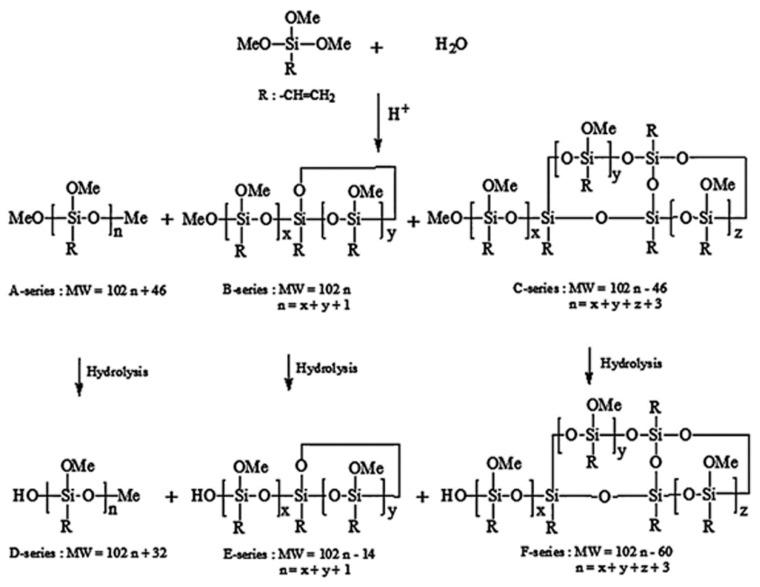
Schematic diagram to show the oligomerization steps. Reprinted with permission from [58]. Copyright Elsevier, 2015.

The formation of silsesquioxanes from organotrialkoxysilanes depends on many parameters, e.g., temperature, water/silane molar ratio, solvent, the concentration of silane, organo groups, and catalysts [22,24,29,56,58,88,90,91]. The conditions to obtain certain silsesquioxanes are still not clear, and many were obtained through trial and error. The suggested reaction schemes are shown in Figure 18, but these vary slightly from one group to the other.

**Figure 19:**
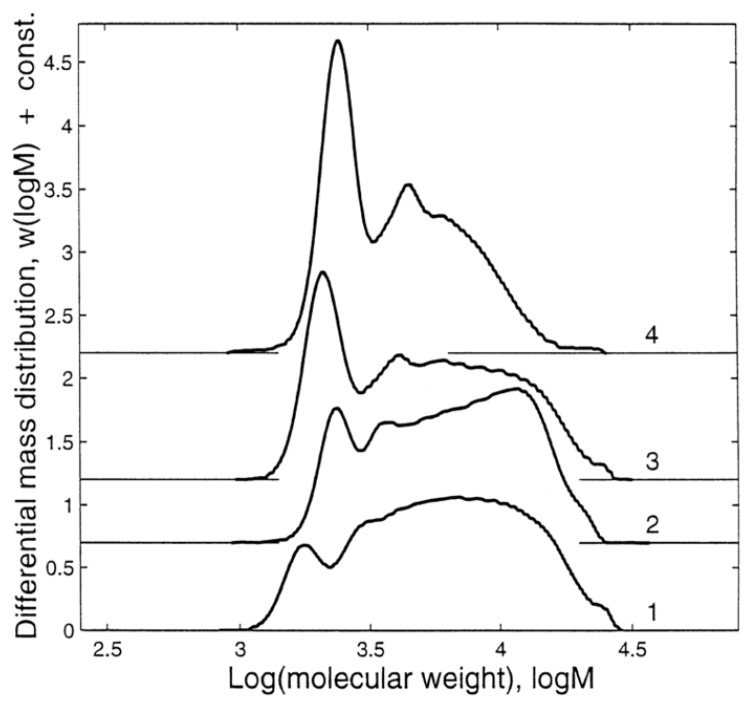
GPC chromatograms of GPS polymerization mixtures catalyzed by DBTDL at different conditions. (1) Volume fraction of DBTDL (*C*) = 1, water/silane ratio (*r*) = 1.5, *T* = 80 °C, time (*t*) = 5 days; (2) *C* = 1, *r* = 5, *T* = 80 °C, *t* = 5 days; (3) *C* = 0.5, *r* = 1.5, *T* = 110 °C, *t* = 14 days; (4) *C* = 0.5, *r* = 5, *T* = 110 °C, *t* = 14 days. Reprinted with permission from [22]. Copyright Elsevier, 2000.

**Figure 20:**
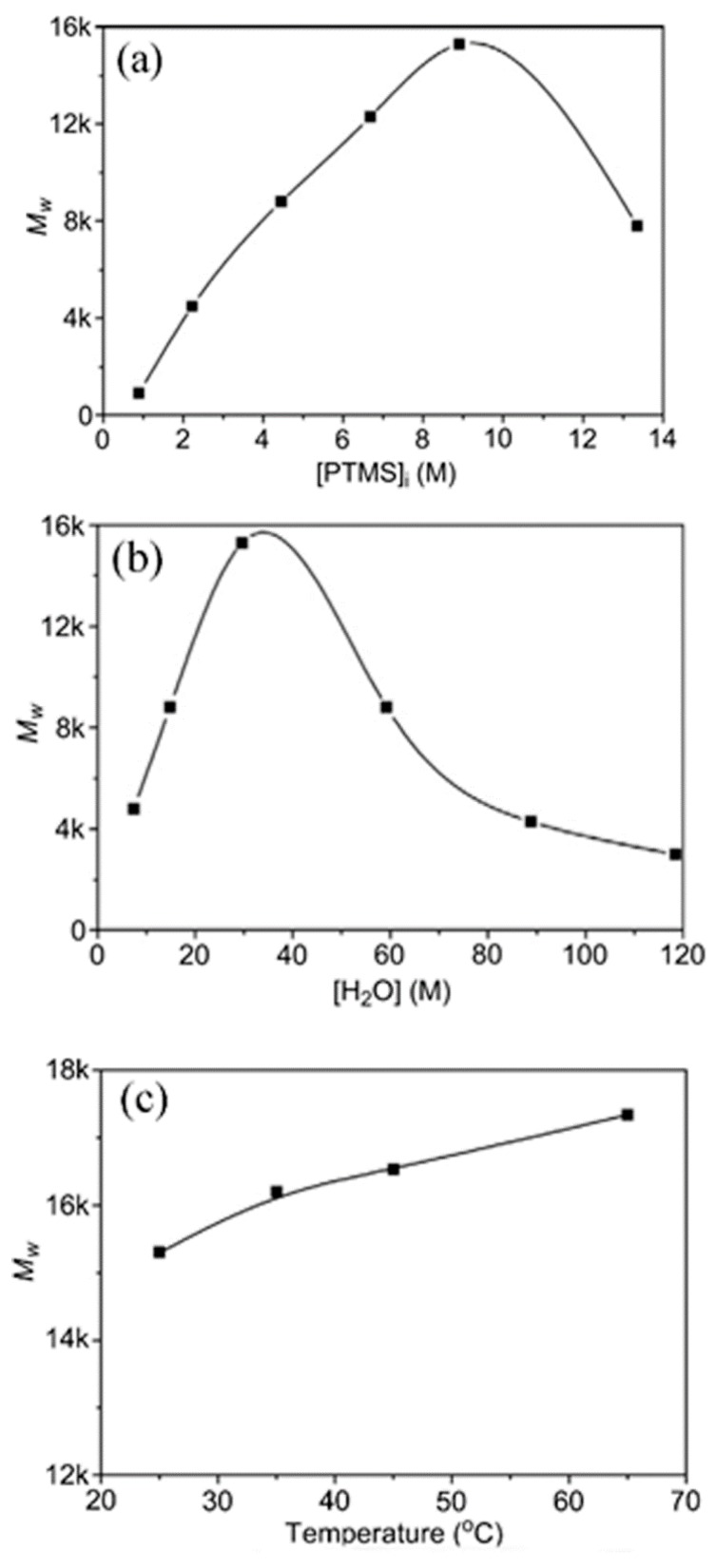
Molecular weight of produced phenyl silsesquioxanes with (**a**) initial concentration of PTMS, (**b**) water concentration, and (**c**) temperature of the reaction medium. Reprinted with permission from [29]. Copyright Elsevier, 2016.

POSS is generally obtained in dilute solutions at a low temperature, while the ladder structure is obtained at elevated temperatures [29]. The polymerization starts with the formation of a small oligomer and then proceeds to high molecular weight oligomers until the gelation point, depending on the water/silane molar ratio, the silane concentration, and the reaction time. It was shown that the organo group had a direct influence on the cubic octamer formed silsesquioxanes [29]. The composition of the silsesquioxanes in the reaction medium changes over time until phase separation (Figure 19). Figure 20 shows the effect of silane and water concentrations, and temperature, on the molecular weight of the silsesquioxanes.

**Figure 21:**
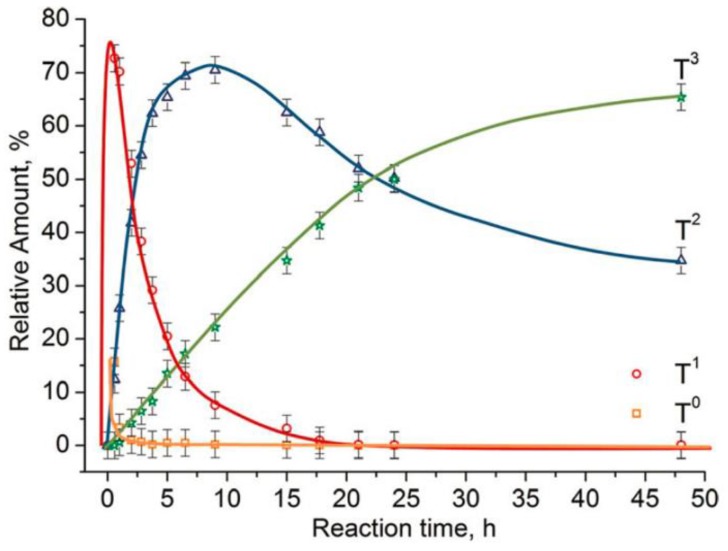
Evolution of the amount of T*^n^* species of MRPMS at 100 °C by using NMR. Reprinted with permission from [24]. Copyright John Wiley and Sons, 2016.

As mentioned before, the trifunctional silanes (T) are defined as T^0^, T^1^, T^2^, and T^3^ species, where the superscripts are the number of siloxane bonds around the central atom. By using characterization techniques, especially NMR, the structure of the produced silsesquioxane can be determined from the ratio of these species. The linear/branched chain silsesquioxanes consist mainly of T^1^ and T^2^, while the cyclic oligomers consist of T^2^. However, the cages and ladder structures are formed from T^3^. Figure 21 shows the development of these species over time.

This section showed that the sol-gel chemistry of silica polymerization were well studied by two different groups. Material scientists studied sol formation to obtain monodispersed silica particles. Their work resulted in the Bogush equation, which can be used to predict the parameters needed to produce silica particles with certain diameters. Geologists and some biologists, on the other hand, studied the formation of silica gel in nature. Most of their work concentrated on tetra-functional silanes (Q-species), but the T-species received little attention. As far as silsesquioxanes (POSS) are concerned, there is no description of well-defined experimental conditions to obtain a certain pre-determined structure.

## 5. Identification Techniques

Many techniques are used to follow the kinetics of the polymerization of silane. The choice of equipment depends on its availability and on which stage of the polymerization should be monitored. There are non-destructive techniques that do not need complicated sample preparation, and these are preferred in the determination of the polymerization kinetics. Nuclear magnetic resonance (NMR), Fourier transform infrared (FTIR), Raman, and small angel X-ray diffraction (SAXS) are examples of such non-destructive techniques. On the other hand, liquid and gas chromatography (LC and GC, respectively) are destructive techniques and need special sample preparation, and should be connected to a mass spectrometer (MS).

### 5.1. NMR Spectroscopy

Most of the kinetics studies of silanes were conducted by using NMR [26,27,29,45,53,60,62,63,65], because NMR signals can be measured in different phases (aqueous or organic) and states (liquid, gel, or solid) without interrupting the reaction. The NMR signals for the tetra-functional silanes (TEOS and TMOS) were discussed in detail [4]. In this work, they identified the signals of each species of hydrolysis and condensation of both silanes (Table 5). There is also a literature review by Miller and coworkers on the use of NMR to study the gelation kinetics of TEOS [11].

**Figure 22:**
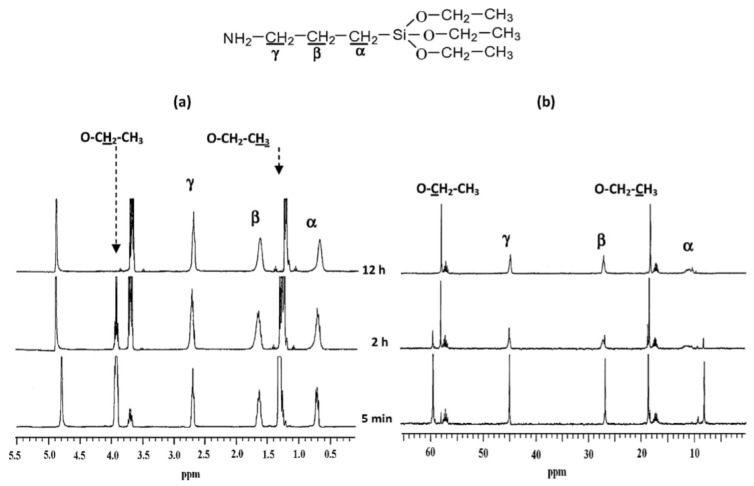
(**a**) ^1^H and (**b**) ^13^C NMR spectra of γ-aminotriethoxysilane (APS) hydrolysis (*t* = 5 min and *t* = 4 h) under natural pH. Reprinted with permission from [49]. Copyright Elsevier, 2013.

It was observed that a change in the leaving group affected the ^29^Si chemical shifts (in ppm) of TMOS and TEOS. It is therefore expected that the addition of different organo-groups will lead to changes in the values of the chemical shifts for the corresponding species. There is, however, little difference in the chemical shifts of the ^29^Si NMR signals for each silane species according to the organo-group. In general, T^0^, T^1^, T^2^, and T^3^ are, respectively, located around –40, –50, –60, and –70 ppm, which are lower than the corresponding species of the tetra-functional silanes, such as TEOS or TMOS [22,60,65]. These signals shifted to higher values in the presences of double bonds or aromatic rings close to the silicon atom, as in the case of vinyl trimethoxy silane [58] and phenyl triethoxy silane [21,56]. Although most of the silane polymerization studies were conducted by using ^29^Si NMR, many authors used ^1^H and ^13^C NMR to conduct studies [19,26,27,49,65]. Some of them followed the reaction by monitoring the ethoxy and ethanol peaks (Figure 22). The main disadvantage of the ^1^H and ^13^C NMR is that one cannot follow the formation of the oligomers as is possible with ^29^Si NMR. 

### 5.2. FTIR and Raman Spectroscopy 

The FTIR spectrum is divided into three regions, that are the far (20 to 200 cm^−1^), the middle (400 to 4000 cm^−1^), and the near (above 4000 cm^−1^) infrared regions. Most papers used the middle infrared (MIR) to study the chemical structure of the compounds, because it has a fingerprint region for any compound. However, there are some kinetics studies that were conducted by using near infrared (NIR) instruments [37]. The use of FTIR and Raman spectroscopy in the kinetics studies of TEOS and TMOS was previously discussed [4], but the focus was mainly on Raman spectroscopy. They showed a comparison between Raman peaks in the range of 300 to 900 cm^−1^ and ^29^Si NMR peaks in the range of −70 to −120 ppm. They assigned the Raman peaks in this region to specific species of TMOS. Zerda et al. [34] tried to estimate the radius of the formed particles by measuring the Raman intensity at 830 cm^−1^, using the equation, I830 cm−1α (83)πr3−2πr2, with methanol as the internal standard.

Usually, FTIR is used in association with other techniques, especially NMR. However, in some cases, FTIR was used alone to conduct kinetics studies [28,87,92]. The disappearance of Si-O-R and the formation of Si-OH and then Si-O-Si or Si-O-M (metal) are normally monitored through FTIR (Table 6). The other peaks (e.g., C≡N, C=O, N-H) are used as an internal reference [92]. In addition to the ability to determine the kinetic parameters of hydrolysis and condensation of silane, the FTIR is used to study the formation of a silane layer over metals (e.g., 3-aminopropyl triethoxy silane cover on copper metal [49]). Moreover, despite the greater difficulty of the identification of silsesquioxanes by infrared, two groups reported FTIR peaks corresponding to some silsesquioxanes (Table 6) [24,59] by relating the FTIR peaks to the corresponding ^29^Si NMR peaks.

**Figure 23:**
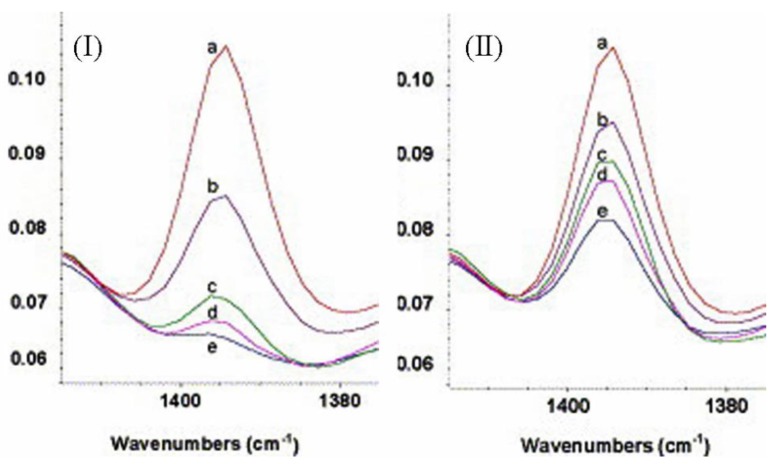
DRIFT spectra of 4-TBN on silica gel (a) at zero time after adsorption; (b) after 1 day; (c) after 3 days; (d) after 6 days; (e) after 8 days at room temperature and (**I**) water-saturated atmosphere, and (**II**) vacuum conditions. Reprinted with permission from [92]. Copyright Elsevier, 2005.

Péré et al. [92] used diffuse reflectance infrared Fourier transform (DRIFT) and transmittance IR to investigate the grafting of a silica gel surface with 3-cyanopropyl triethoxysilane (4-TBN) under different conditions. They used 4-TBN due to the presence of the cyano group, which has a strong and stable peak at 2253 cm^−1^, and they used transmittance IR to determine the amount of 4-TBN, which adsorbed physically onto the surface of the silica, so that they could determine the 4-TBN that was chemically bonded to the surface. They used DRIFT at 1394 cm^−1^ (bending of CH_3_ of the ethoxide group) to monitor the reaction rate of the chemical bonding of 4-TBN to the surface of silica gel (Figure 23).

It was shown [4] that the Raman and ^29^Si NMR spectra for TMOS complemented each other well, but there was no mention of organo-functionalized silanes. Table 7 represents the Raman peaks for the tetra-functional silane species. The region between 200 and 1500 cm^−1^ is the most useful region to study the kinetics of silanes, since there is a lot of interference in the higher wavenumber region [54] (Table 8).

Ferri et al. [54] used Raman spectrometry to monitor the hydrolysis and condensation of octyl triethoxysilane (OES)/TEOS in real time during the coating process. They investigated the hydrolysis by monitoring the disappearance of peaks at 650 and 810 cm^−1^, which represent the Si-O breathing and CH_2_ rocking of alkoxide, respectively, as well as the appearance of the peaks at 710 and 881 cm^−1^ related to the formation of hydrolyzed silane and ethanol, respectively. The condensation reaction was followed by monitoring the broad peak between 250 and 500 cm^−1^ due to Si-O-Si bond bending, and the peak at 840 cm^−1^ due to Si-O-Si bond stretching.

### 5.3. Mass Spectroscopy (MS)

MS is a technique used to determine the molecular weight and mass fragments of the chemical compounds with different ionization methods. There are different types of mass spectrometers (e.g., single quad, triple quad, time of flight (TOF), magnetic sector). In addition, there are two types of ionization: Hard and soft ionizations. Hard ionization is electron ionization (EI), while soft ionization is electrospray ionization (ESI), chemical ionization (CI), or photochemical ionization (PCI). Usually, the MS is combined with other instruments, such as liquid chromatography (LC), gas chromatography (GC), matrix-assisted laser desorption/ionization (MALDI), and inductively coupled plasma (ICP). The EI is normally used with GC-MS, while the ESI is used with LC-MS and MALDI with TOF. MS was initially used to monitor the small molecules at the start of a reaction, especially in the case of studying Q silanes. Recently, MS is used to study silsesquioxanes due to the formation of oligomers that are smaller than macromolecules. The use of MALDI-TOF to screen macromolecules increases the benefits of using MS in sol-gel systems. 

The use of MS to study the hydrolysis and condensation of metal alkoxides (e.g., silicon alkoxide) was discussed previously [10]. In our work, we try to discuss the principles, which will help in using the technique, as well as TOF, which is not discussed in the mentioned publication. Two conditions guide us in using MS, the boiling point and thermal stability of the silanes and their species in the reaction medium. If they are thermally stable and volatile, one should use GC/MS, which gives good separation and a rich MS spectrum [36,93]. However, GC/MS is not a common technique due to the use of water as a reactant. Otherwise, one should use quad-MS or TOF-MS, depending on the mass/charge ratio (*m*/*z*) of the compounds. If the *m*/*z* < 2000 amu, then LC-MS should be used [56,58,91]. If it is more than 2000 amu, the siloxanes should be investigated by MALDI-TOF [88,94] or LC-TOF [21,89].

**Figure 24:**
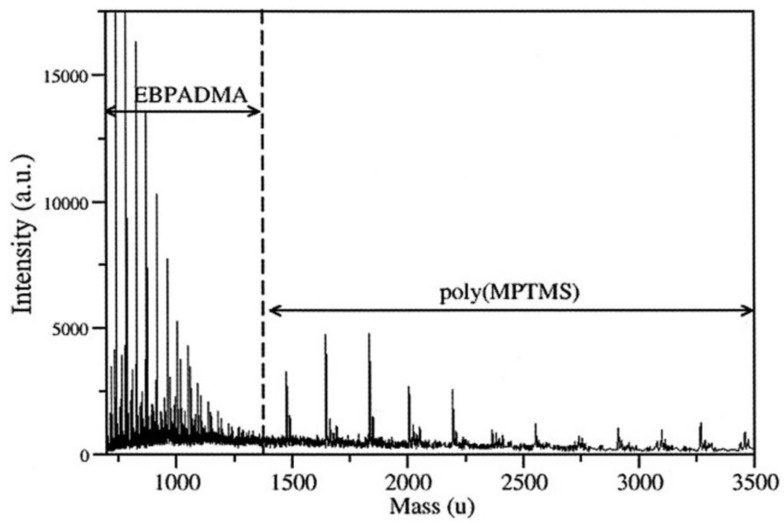
The mass spectrum of the polymerization of MPTMS with dental monomer (ethoxylated bis-phenol A dimethacrylate (EBPADMA)). Reprinted with permission from [94]. Copyright John Wiley and Sons, 2005.

MALDI-TOF is used to determine the produced oligomers or macromolecules in real time, where the reactants with a concentration of 45 mg L^−1^ are mixed with the MALDI matrices (e.g., 2,5-dihydroxybenzoic acid, sinapinic acid, etc.). A laser beam with energy around 5 µJ is then used to ionize all the compounds in the reaction medium. MALDI-TOF was used to investigate the reaction of γ-MPS in various dental solutions [94] to differentiate between the produced oligomers due to the polymerization of the dental monomers and MPTMS. It was shown that there is no evidence of an interaction between the dental monomers and MPTMS. Dental oligomers were observed below 1475 amu, and silsesquioxanes between 1475 and 9000 amu (Figure 24). LC-TOF is used when we need to separate the produced oligomers and investigate each one individually. 

### 5.4. Chromatography

The spectroscopy techniques (NMR, FTIR, MS, etc.) are very important and give valuable information about the kinetics of a reaction, but they cannot give information about the individual species of silane inside the reaction medium. One should therefore use a separation technique, which could be LC, GC, or gel permeation chromatography (GPC). Each technique has its own instrument configurations, the discussion of which is out of the scope of this review. As mentioned before, GC is used with volatile and thermally stable silane and LC is used with non-volatile and thermally unstable compounds, while GPC is used to determine the molecular weight distribution of oligomers by using external standards. The main difference between GC and LC is the mobile phase; in GC, it is a gas (e.g., helium, hydrogen, and nitrogen), while in LC, it is a liquid (e.g., water, ethanol, methanol, acetonitrile, hexane, etc.).

#### 5.4.1. Gas Chromatography (GC)

**Figure 25:**
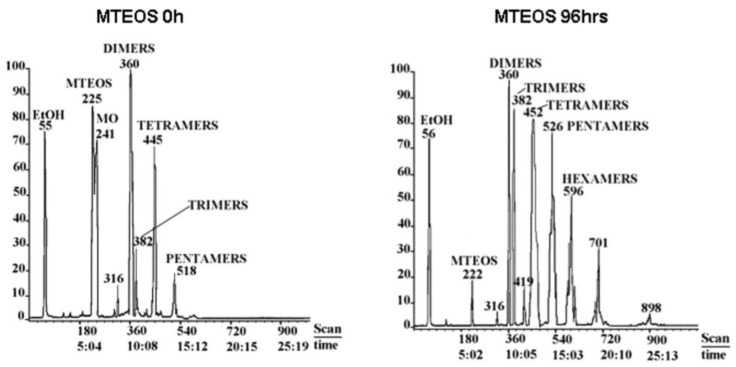
The GC/MS chromatograms of methyl triethoxysilane and its species in the presence of alcohol, water, and HCl. Reprinted with permission from [10]. Copyright John Wiley and Sons, 2016.

Because the use of GC is limited by the volatility and thermal stability of the compounds, it is used to determine the silane itself or the hydrolysis and early stage condensation products. A good combination between the detectors and GC columns can be used to investigate the kinetics of silane polymerization, especially GC/MS, because it does the separation and identification (Figure 25) [4,10]. Yevchuk et al. [38] used GC with a thermal conductivity detector (GC/TCD) to conduct kinetic studies of the hydrolysis and early stage condensation of TEOS, while Chen et al. [36] used GC/MS and GC with a flame ionization detector (GC/FID) to study the kinetics of the formation of monodispersed silica particles after extracting the TEOS with n-heptane. GC/MS is a very useful technique that can be used to separate, identify, and quantify the materials in solutions, but its running cost is very high.

#### 5.4.2. Liquid Chromatography

**Figure 26:**
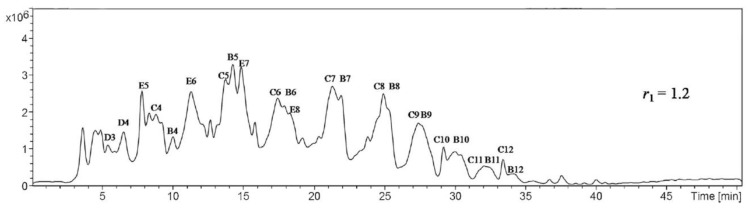
Total ion chromatogram (TIC) of vinyl siloxanes oligomers at a molar ratio (water/silane) equal to 1.2. Reprinted with permission from [58]. Copyright Elsevier, 2015.

GC is only suitable to study the hydrolysis trends and not the oligomers’ distribution, because the investigation of the oligomers by GC requires their silylation by alkyltrichlorosilanes, which produces a side reaction that interferes with the investigated silicate species [56]. Liquid chromatography can be used without derivatization (silylation) in the presence of any quantity of water and other solvents. If the silane compounds have ultraviolet (UV) activity, it can be determined by using high-pressure liquid chromatography (HPLC)/UV; LC-MS, on the other hand, is suitable for all silanes due to its universal detector. The separation normally occurs due to the distribution of the analytes (silanes) between the mobile and stationary phases, depending on the hydrophobicity of the analytes. Different high-pressure liquid chromatography (HPLC) columns can be used to separate the silanes, e.g., C8, C18 [56,58], NH_2_, cyano, or phenyl. Two different groups separated the oligomers of vinyl and phenyl silanes by, respectively, using LC-MS and C18 columns. One group [50] divided the produced phenyl oligomers into three groups (A, B, and C), while another group used six groups of vinyl oligomers to (A, B, C, D, E, and F) [56,58]. All these could be identified by using LC-MS (Figure 26).

#### 5.4.3. Gel Permeation Chromatography

GPC (SEC or GFC) is similar to LC in the instrumentation and technique, and the only difference is the separation principle. In regular LC, the separation depends on the hydrophobicity, but in GPC, it depends on the molecular size. Many articles reported the use of GPC to separate silane oligomers [21,22,24,59,89,90]. However, the use of LC-MS is probably better than GPC because the polymerization of trialkoxysilanes produces small silsesquioxanes less than 9000 amu, with the majority of them below 2000 amu [94]. GPC, on the other hand, is used for a wide range of polymer sizes (200 to 10000000 amu), and therefore the separation needs a variety of GPC columns [59] depending on the expected molecular weight. These columns are much more expensive than regular HPLC ones. The comparison of Figure 19 and Figure 26 clearly shows the difference between LC and GPC. However, GPC is used in many applications, such as the separation of cage-like GPS silsesquioxanes under different conditions [22].

### 5.5. Other Less Common Techniques 

In addition to the previous techniques, many other instruments, such as XPS [45,47], small angel X-ray spectroscopy (SAXS) [42], calorimetry [30,40], conductivity [2,36,43,50], colorimetry [32,33,40], transmission electron microscopy (TEM) [43,51], atomic absorption spectrometry (AAS) [43], titration [51], micro-pycnometry [51], ICP-AES [33], and dynamic light scattering (DLS) [95,96], were used to investigate the polymerization kinetics of silanes and its products. Lindberg’s group introduced a new method to follow the hydrolysis and condensation of octyl triethoxysialne over the surface of a water droplet in an octane solvent by surface tension [25]. The gelation time of the polymerization of TMOS in an acidic medium at different conditions was determined by using rheological and acoustic techniques [85].

Spectroscopic techniques (especially NMR) were used extensively to study the polymerization kinetics of silanes, because it is a simple and non-destructive technique. However, it has limitations in that it cannot separate the reactive species, it is expensive, and its running cost is high. Chromatographic techniques can be used to overcome the limitation of separating the reactive species.

## 6. Theoretical Studies

Many parameters control the polymerization reaction of silanes, and this requires significant experimental efforts to study the effect of each parameter on the polymerization process. Methods should therefore be found to help in understanding and designing the polymerization reaction theoretically, taking into account the different requirements. We can use modeling, experimental design, or computational chemistry. The modeling and experimental design are beyond the scope of this review, because they are generic tools, but we shall look at computational chemistry, dealing with the chemical structures of the reactants, intermediates, and products.

Computational chemistry methods try to predict the energy of a chemical system depending on different principles. From these energies, the possibility of a reaction and its preferred pathway can be predicted. The computational methods are molecular mechanics (MM), quantum mechanics (QM), ab initio, molecular dynamics (MD), Monte Carlo (MC), and semi-empirical. Which method is used depends on its accuracy and computational time. These methods, and others, have already been explained in a number of texts [15,97]. Most researchers in the field of silica used the ab initio [1,17,18,70] and DFT [3,35,71,98,99,100] methods. The ab initio method is more accurate, but takes a long computational time, while the DFT method gives acceptable accuracy with moderate computational time. 

Computational chemistry is mainly used to predict the mechanisms of a reaction and the preferred pathways. There are, however, a few studies about the kinetics of the reaction. Most of this computational chemistry research was conducted on hydrolyzed and non-hydrolyzed tetra-functional silanes (Q), while the mechanisms of the polymerization of the organo-silanes, but not the kinetics, received some attention. Examples are the use of the ab initio method with bases (RHF/3-21G(*), RHF/6-31G*, and RHF/6-31G*) to study the polymerization of methyl trimethoxy silane [1], while the DFT method was used to study the interaction between MRPMS and silica nanoparticles under acidic conditions [71]. 

**Figure 27:**
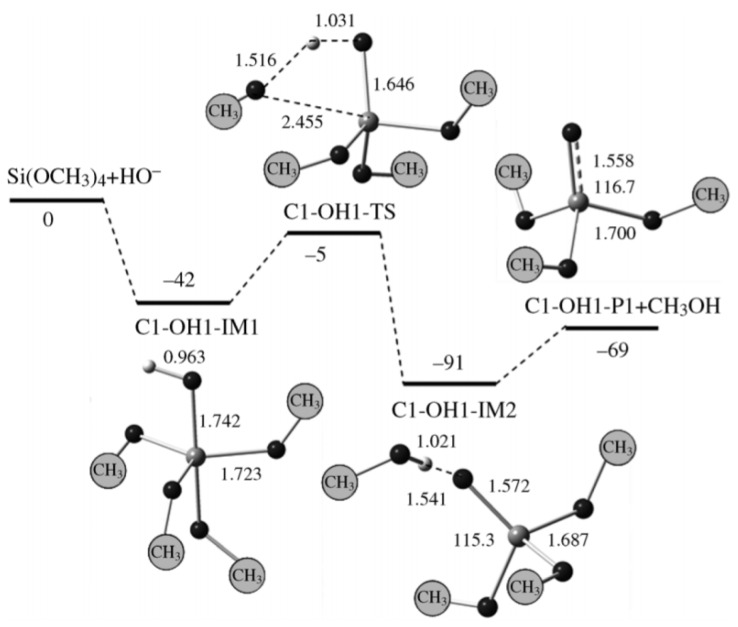
Propyl ethoxy silane (PES) of the first order hydrolysis of TMOS in an alkaline medium. Reprinted with permission from [35]. Copyright John Wiley and Sons, 2012.

In this work, we shall mainly focus on the research by Cheng and co-workers [35,98,99,100], where the hydrolysis and condensation of TMOS under different conditions (e.g., neutral, acidic, alkaline, and the presence of fluoride (F^−^) ions), as well as the oligomerization of dihydroxy silicone (SiO(OH)_2_), was investigated. This was compared with the hydrolysis and condensation of TEOS, trimethoxy aluminumate Al(OCH_3_)_3_, and tetramethyl titanate (Ti(OCH_3_)_4_). They used the DFT method, where the molecules were fully optimized at the B3LYP/6-31G(d,p) level, followed by single-point energy (SPE) calculations and entropy calibrations at a higher B3LYP/6-311++G(d,p) level, combined with a conductor-like polarizable continuum model (CPCM). The CPCM was used to consider the solvent effect. In some cases, they used M06-2X/6-311++G(d,p) for comparison with B3LYP. Eventually, they used B3LYP in the rest of their work. Their results can be summarized as follows:

- The structure of the TMOS with one water molecule is a trigonal bipyramid in neutral medium, while it is octahedral in alkaline and acidic media.
- Increasing the number of the water around the silane (e.g., TMOS) decreases the energy barriers of the transition state due to a lowering of the hydrogen bond strain.
- The potential energy surface is composed of stationary points (e.g., reactant complex (C)—intermediate (IM)—transition state (TS)—intermediate 2 (IM2)—product complex (P)) (Figure 27).
- The water molecule will attach from the front in a neutral medium, while it will attach from the back in alkaline and acidic media. It links to the oxygen atom in a Si-O bond through hydrogen bonding.
- The effect of the solvent on the energy barriers for the first, second, third, and fourth orders of TS (TMOS) is as follows: Pure methanol (283.6, 276.3, 259.0, and 259.1 kJ mol^−1^), water (119.7, 121.7, 123.0, and 123.0 kJ mol^−1^), and water-methanol mixture (121.7 125.5, 126.9, and 125.1 kJ mol^−1^), respectively. The reaction in pure methanol leads to the formation of silicone and not hydrolyzed silane at high temperatures. Methanol was found to have a small effect on the hydrolysis in the presence of water.
- The effect of attacking ions (neutral, OH^−^, H^+^, and F^−^): The energy barriers for TMOS in a neutral medium are 119, 106, 109, and 109 kJ mol^−1^. In an acidic medium, they are 52, 61, and 69 kJ mol^−1^. The hydrolysis in the acidic medium is incomplete due to proton blocking, so the last methoxy group is very difficult to hydrolyze. In an alkaline medium, they are 77, 157, 155, and 162 kJ mol^−1^. For the fluoride (F^−^), they are 84.2, 88.5, 77.2, 81.9, and 82.2 kJ mol^−1^ (before entropic correlation).
- In terms of the effect of the leaving group, the energy barriers for the hydrolysis of TMOS are 119, 106, 109, and 109 kJ mol^−1^, while for TEOS, they are 120,106, 123, and 109 kJ mol^−1^. They attributed the little difference in the energy barriers to a steric effect.
- They discussed the dimerization between various hydrolyzed TMOS and they reported that hydrolysis predominates energetically in a neutral medium. In an acidic medium, the dimerization energy barriers are larger than the hydrolysis ones, so the hydrolyzed forms are predominated in the solution. In an alkaline medium, they reported that the dimerization occurred by an SN1 mechanism with an energy barrier of 46 kJ mol^−1^ between the two first orders of TMOS hydrolyzation. F^−^ alters the dimerization mechanisms, which lie between SN2 and SN1, and the fluoride ion attacks the silicon atom in a way similar to the hydroxide ion.
- Oligomerization of the simplest silicone (SO(OH)_2_): Here, they studied all kinds of oligomerization addition between monomers and oligomers up to tetramers. They found that the hexagonal and octagonal trimer and tetramer with two hydroxyl groups each are more stable. In the case of cyclic oligomers, the hydroxide hydrogen can swing freely due to low energy barriers. All the oxygen atoms participated in the formation of conjugated π bonds, which stabilized the ringed oligomers.

**Figure 28:**
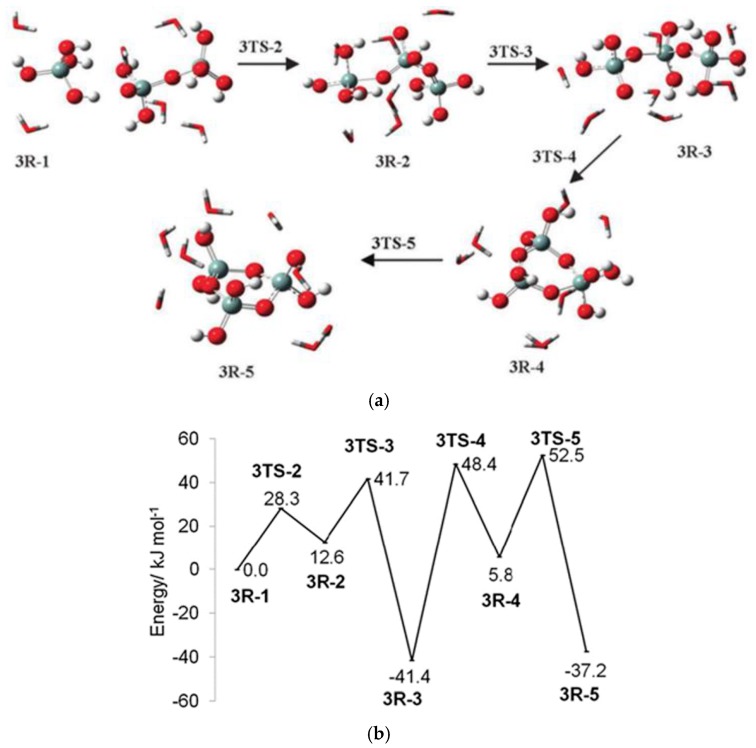
(**a**) Scheme of the H_4_SiO_4_ + H_5_Si_2_O_7_^−^ channel for the formation of a trimer, (**b**) the PES for this channel. Reprinted with permission from [17]. Copyright Royal Society of Chemistry, 2013.

McIntosh and co-workers published three articles about the oligomerization of silicic acid in an alkaline solution by using the MP2/6-31+G(d)//HF/6-31+G(d) level of the ab initio theory and including CPCM-SPE corrections. The first paper [17] discusses the formation of different species of silicic acid (trimers and tetramers) and their energy barriers. They presented the previous work in their introduction, and the limitation of using implicit water. They found that the energy barrier for the formation of a small oligomer is 56–59 kJ mol^−1^, while for a large oligomer it is 30–31 kJ mol^−1^, and for the elimination of water from the condensation it is 54–56 kJ mol^−1^. They then used six or eight molecules of explicit water in their calculation, and they found that it is extremely important in the stabilization of the intermediate and transition states, compared to the case where they used implicit water. All monoionic channels for the formation of linear/branched or cyclic trimers and tetramers were investigated (e.g., the H_4_SiO_4_ + H_5_Si_2_O_7_^−^ channel for the formation of trimers) (Figure 28). Moreover, they compared the obtained values with those values in the previous literature. They stated that the use of energy barriers can explain the mechanisms, but they could not give a full picture of the silicate growth model because the reaction is time, concentration, and pH dependent, and the reaction channels are highly interconnected.

Because of the limitations in the first paper [17], they published a second paper in which they tried to obtain the kinetics parameters (e.g., rate constant) theoretically [18]. They computed the rate constants by using a generalized transition state theory rate constant equation (Equation (23)):

(23) $$k\left(T,s\right)=\kappa \left(T\right)\frac{k_{B}T}{h}\;\frac{Q\left(T,s\right)}{\Phi \left(T\right)}\;e^{\frac{-V_{MEP\left(s\right)}}{k_{B}T}}$$

where κ(T) is the transmission coefficient, and $k_{B}\;\mathrm{and}\;h$ are the Boltzmann and Planck constants, respectively. Q(T,s) is the transition state quasi-partition function (the imaginary frequency has been projected out). Φ(T) is the partition function of the reactants and $-V_{MEP\left(s\right)}$ is the energy barrier at the minimum energy path (MEP) for any stationary point(s). They also wrote a paper on the effect of the temperature and pH on the rate constants of silica growth by using the ab initio and DFT methods combined with the transition state theory [72]. Zhang and co-workers performed a complete investigation on the initial stages of silicate oligomerization mechanisms and kinetics. They used the off-lattice kinetic Monte Carlo (continuum kMC) method, where they obtained the required parameters for the kMC by using the DFT theory [73]. Using the ab initio, DFT, MC, or MD approaches individually will limit a complete description of the silicate chemistry, so the best way is to have a combination of the ab initio or DFT methods with the MC, transition state theory, or MD approaches. These works are valuable in the prediction of the kinetic parameters of some species (e.g., dimer, linear, cyclic trimer formation), and are aligned with experimental data. Although it shows the effect of temperature and pH on the species’ concentration, one still needs to improve these kinetic models by using experimental data or by refining some of the approximations in the model, as suggested by McIntosh [18].

## 7. Conclusions

The polymerization of silane is a stepwise composite reaction, which consists of hydrolysis, condensation, and phase separation reactions. Each step in these reactions has its own kinetics. There are many parameters that control the overall kinetics of the reaction. These parameters are the catalysts, molar water to silane ratio (r), temperature, pH, and ionic strength (IS). In general, the hydrolysis is fast, and the condensation is slow in acidic media, while the opposite is true in an alkaline medium. The reaction order is first order with respect to silane, but the researchers did not agree on the order with respect to water. Most researchers did not mention the order with respect to alcohol, except for one group who reported it as first order with respect to ethanol [27]. This is, however, not in line with the Le Chatelier principle, because the ethanol is used as a solvent (excess amount) in the sol-gel process. 

The oligomerization of silane begins with the formation of dimers, trimers, and tetramers, and then proceeds to form sols, gels, or silsesquioxanes depending on the silanes and water concentration, temperature, and other parameters. The cyclization depends mainly on the water concentration, while the silsesquioxane structures (POSS, ladder, and random) depend on the temperature of the reaction and the concentration of silanes, in addition to the catalyst in some cases. The growing of silane oligomers depends mainly on time. The Stöber method is used to prepare monodisperse silica particles in an alkaline solution, which depends mainly on the concentration of TEOS, ammonia, and water. 

Many techniques are used to follow the reaction kinetics of silanes. The spectroscopy techniques, which are very useful, cannot differentiate between the silane species in the reaction medium, while the chromatographic methods can do that, but they are time-consuming techniques.

Despite a number of efforts to study the kinetics of silanes polymerization, a number of issues are still unclear due to the large number of parameters that can influence the properties of the final products at different stages of the preparation. An example is where silica nanoparticles were prepared under identical conditions, but dried under different conditions, and particles with different surface areas and pore sizes were obtained [101]. Researchers therefore know the trend of silane polymerization qualitatively, but to obtain a comprehensive image with quantitative details requires a lot more work. Hence, theoretical studies are becoming very important, especially computation chemistry. To study the reaction mechanisms, the DFT or ab initio methods can be used, but the DFT method is the better choice due to its good accuracy. The kinetic and thermodynamic parameters can be obtained by using molecular dynamics or Monte Carlo (MC) with the DFT or ab initio methods. 

## Figures and Tables

**Figure 4:**
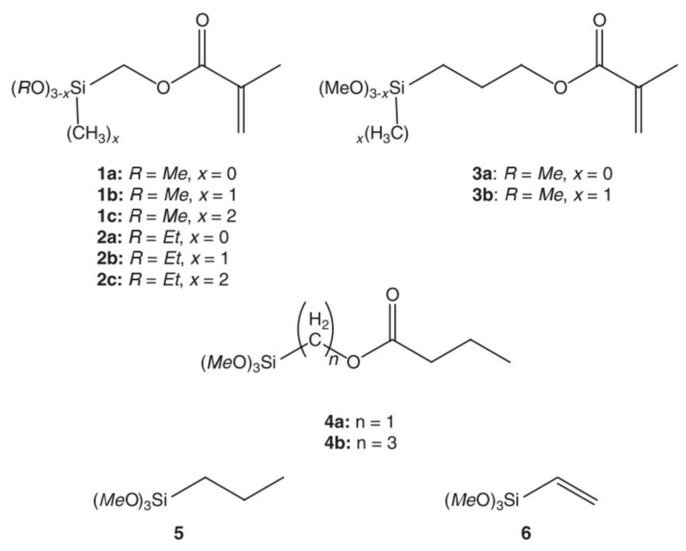
Chemical structures of the molecules used by Altemann et al. Reprinted with permission from [46]. Copyright Springer Nature, 2003.

**Table 1:** Kinetics information for the polymerization of alkoxysilanes and organoalkoxysilanes.

Precursor	Reaction Conditions	Values			Used Techniques	Reference
TEOS	They used Stӧber’s conditions with variation in ammonia and water concentrations. The concentration of TEOS was kept constant at 0.5 M. The solvents used were ethanol and methanol.	They determined the hydrolysis rate constants of the TEOS hydrolysis steps (Figure 3). The overall hydrolysis/condensation rate constants for the second, third and fourth hydrolysis steps were also reported. The hydrolysis of the second step was faster than that for the first step under all conditions. The first and second hydrolysis rate constants were 3.91 and 78.0 × 10^−4^ M^−1^ min^−1^ in ethanol, but they were 22.24 and 88.3 × 10^−4^ M^−1^ min^−1^ in methanol under the same conditions.			NMR 300 and 400 MHz. SAXS	[42]
TEOS	The TEOS was hydrolyzed by using phosphoric acid in water at different TEOS:water:phosphoric ratios and different temperatures.	The hydrolysis rate constants ranged from 1.1 to 5.4 × 10^2^ M^−1^ sec^−1^. The calculated activation energy with a 2.0:0.5:0.05 mL ratio under the reaction conditions was 33.3 kJ mol^−1^.			GC/TCD	[38]
TEOS	Hydrolysis of TEOS in acidified water with hydrochloric acid and ultrasonic power.	The hydrolysis rate constant ranged between 4.5 and 65 × 10^−2^ M^−1^ min^−1^, depending on the concentration of the acid.			Calorimetry	[40]
TEOS	Bogush et al. studied the hydrolysis and condensation reactions of TEOS under different conditions in alkaline medium (Stӧber method).	They assumed that all the reactions were first order. The hydrolysis rate constants ranged from 1.4 to 8 × 10^4^ s^−1^ depending on the concentration of water and ammonia. The average condensation rate constants ranged from 3.2 to 32 × 10^3^ s^−1^, also depending on the concentration of water and ammonia. The hydrolysis was not noticeably affected by the addition of sodium chloride, while the condensation rates decreased with the addition of the salt.			Using different techniques such as TEM, NMR, conductivity, and atomic absorption spectrometry (AAS)	[43]
TEOS	Hydrolysis of TEOS in acidified water by using ultrasound at a constant ultrasonic (US) power of 60 W and different pH (0.8 to 2).	The hydrolysis rate constant was calculated at all the pH values by the following equation: 6.1 × [H^+^] M^−1^ min^−1^ at 39 °C, where [H^+^] is the concentration of proton.			Calorimetry	[41]
TEOS	Hydrolysis of TEOS in acidified water without homogenizing agent and by using different ultrasound power.	The hydrolysis rate constants ranged from 2.8 to 5.8 × 10^−3^ M^−1^ sec^−1^ depending on the power of the ultrasonic bath.			Calorimetry	[30]
TEOS	The hydrolysis rate constants of TEOS in acidic medium (<0.003 M HCl) and alkaline medium (0.04 to 3 M NH_3_).	The activation energy in the acidic medium ranged from 11 to 16 kcal mol^−1^. The hydrolysis rate constant ranged between 0.002 and 0.5 M^−1^ h^−1^ depending on the ammonia concentration. The activation energy in basic medium was reported to be 6 kcal mol^−1^.			NMR	[6]
OTES	Hydrolysis (*k*_h_) and condensation (*k*_c_) constants of octyl triethoxy silane (OTES) between two liquids (octane and water) at different OTES concentrations (0.001, 0.1, and 0.1 M).	Conc. of OTES (M)	0.001	0.01	Measuring of interfacial tension between the octane and water layers by using the pendant drop method.	[25]
		*k*_h_ (min^−1^)	0.6	0.7		
		*k*_c_ (m^2^ mmol^−1^ min^−1^)	72	82		
OTES	Hydrolysis of OTES in liquid carbon dioxide and near supercritical conditions.	The hydrolysis rate constant was 0.0426 min^−1^			TGA and NMR	[44]
APTS	Hydrolysis of aminotriethoxy silane (APTS) in deuterated ethanol with water/silane ratio = 1 and without catalyst.	The APTS hydrolyzed in two steps, initial and secondary steps with rate constants 2.77 and 0.733 × 10^−4^ sec^−1^, respectively, at 25 °C. The activation energies of the two steps were, respectively, 34.4 and 30.6 kJ mol^−1^.			NMR 300 MHz	[27]
GPS	The hydrolysis of γ-glycidoxypropyl trimethoxy silane (GPS) in different methanol/water ratios and using acetic acid as a catalyst	They graphically presented the concentrations of GPS and its hydrolyzed species vs time, instead of reporting numerical values of their rate constants.			NMR	[45]
GPS	Hydrolysis of GPS in non-aqueous solution (95% ethanol, 1% silane, and 4% water) with organotin and other organometallic catalysts.	The hydrolysis rate constants ranged from 0.01 to 22 × 10^−4^ min, depending mainly on the catalyst, but also on the solvent.			NMR	[26]
PTMS	The phenyltrimethoxysilane (PTMS), propyl trimethoxy silane (PrTMS), and methacryloxypropyltrimethoxy silane (MPTMS) were hydrolyzed in THF and KCO_3_ as catalyst in excess water.	Reaction rate constants (k) of PTMS, PrTMS, and MPTMS were 2.87 ± 0.14 e^−8^ M^−2.3^ s^−1^, 1.26 ± 0.11 e^−8^ M^−2.1^ s^−1^, and 1.42 ± 0.11 e^−8^ M^−1.8^ s^−1^, respectively.			NMR	[29]
Silicic acid	The silicic acid was condensed in aqueous solution under different ionic, pH, and silicic concentrations.	The reaction rate constants ranged from 4.13 to 7.36 × 10^−7^ mmolal^−3^ s^−1^ depending on the ionic strength, pH, and the initial concentration.			UV/Vis spectrometry, ICP-AES	[33]
Ortho-silicic acid	Ortho-silicic acid (10 and 30 mM) was produced from the acid hydrolysis of dipotassium tris(1,2-benzene-diolato-O,O’) silicate. It underwent condensation at different pH (3.4–6.8).	The reaction rates ranged from 1.5 × 10^−8^ to 3 × 10^−6^ mM^−2^ s^−1^ with changing the pH of the reaction medium.			^1^H NMR, UV/Vis spectrometer	[32]
DMDEOS MTES TEOS	The dimethyl diethoxy silane (DMDEOS), methyltriethoxy silane (MTES), and TEOS were hydrolyzed in acidic medium.	The hydrolysis constant of DMSEOS ranged from 0 to 0.6 M^−1^ min^−1^ at pH 2 to 5. The hydrolysis constants of MTES ranged from 0 to 0.23 and for TEOS from 0 to 0.18 M^−1^ min^−1^ at pH 2 to 4. The activation energies of MTES at pH 3.134 and 3.83 were calculated as 57.61 and 97.84 kJ mol^−1^, respectively. The activation energy of TEOS at pH 3.134 was found to be 31.52 kJ mol^−1^.			FT-NIR	[37]
Model polymer	Methoxysilanes-terminated polybutadiene was used as model polymer. It was crosslinked under certain conditions (temperature of 25 °C, humidity of 50%) with different catalysts and water content.	The hydrolysis rate of the model polymer depended on the catalysts and their concentrations. The hydrolysis rate constants ranged from 0.29 to 5.4 × 10^−4^ min^−1^ with different catalysts at a concentration around 3.0 mol %. The hydrolysis rate constants for the mixed catalysts ranged between 2.1 to 5.3 × 10^−4^ min^−1^ depending on the catalyst and co-catalyst combinations.			ATR-FTIR	[28]
Different silanes	Hydrolysis of different silanes that are drawn in Figure 4 under acidic and alkaline conditions	The hydrolysis rates are listed in Section 3.4.2.			NMR	[46]
Different amino-trialkoxysilanes	Hydrolysis of these silanes were conducted by using HCl as catalyst.	The hydrolysis rate for these silanes ranged between 5.5 to 97 mM^−1^ h^−1^ depending on the type of the silane.			XPS	[47]
MTMS	Hydrolysis of methyl triethoxy silanes in different solvents and at different temperatures in alkaline medium.	The hydrolysis rate constant of MTMS in methanol was 2.453 × 10^4^ sec^−1^ at 30 °C, and the activation energy was 50.09 kJ mol^−1^. The hydrolysis rates changed significantly with changing the solvents.			FTIR	[48]

**Table 2:** Hydrolysis rates of GPS with different catalysts. Reprinted with permission from [26]. Copyright Elsevier, 2006.

Catalyst	Solvent	Rate Constant/10^−4^ min^−1^
None	d6-acetone	<0.01
Zinc acetylacetonate hydrate (ZnAA)	d6-acetone	4.3
Zirconium acetylacetonate (ZAA)	d6-acetone	2.0
Aluminium acetylacetonate (AAA)	d6-acetone	6.0
Zirconium tetrakis(2,2,6,6-tetramethyl-3,5-heptadionate) ZTT	d6-acetone	18
Zinc bis(2,2,6,6-tetramethyl-3,5-heptanedionate) ZTH	d6-acetone	0.45
Chromium (III) acetylacetonate	d6-acetone	<0.01
Chromium (III) acetylacetonate (1% by mass)	d6-acetone	<0.01
Zinc acetylacetonate hydrate (ZnAA)	d6-ethanol	4.3
Aluminium acetylacetonate (AAA)	d6-ethanol	18
Zinc bis(2,2,6,6-tetramethyl-3,5-heptanedionate) (ZTH)	d6-ethanol	22
Chromium (III) acetylacetonate	d6-acetone	<0.01
Chromium (III) acetylacetonate (1% by mass)	d6-acetone	<0.01

Mass ratio of solvent:D_2_O:silane:catalyst is 94.96:4:1:0.04 unless otherwise stated.

**Table 3:** Integrated peak areas of phenylsiloxanols prepared at various molar ratios of water to silane agent (*r*_1_), where n is the number of silane monomers in oligomers. Reprinted with permission from [56]. Copyright Elsevier, 2015.

Oligomer	Peak Area (%)									
	*n* = 1	*n* = 2	*n* = 3	*n* = 4	*n* = 5	*n* = 6	*n* = 7	*n* = 8	*n* = 9	Subtotal
*r*_1_ = 0.5										
A-series	1.03	9.20	23.70	31.01	12.01	2.39	-	-	-	79.35
B-series	-	-	2.40	7.13	7.40	3.07	0.65	-	-	20.65
C-series	-	-	-	-	-	-	-	-	-	-
*r*_1_ = 1.0										
A-series	0.66	6.01	19.54	30.19	15.82	4.81	1.25	0.13	-	78.40
B-series	-	-	1.93	7.11	6.43	4.08	1.38	0.31	-	21.23
C-series	-	-	-	-	-	0.29	0.08	-	-	0.37
*r*_1_ = 1.5										
A-series	1.0	3.53	17.5	26.94	15.22	3.91	0.89	0.13	-	69.11
B-series	-	-	1.8	9.75	9.96	5.87	1.85	0.49	-	29.71
C-series	-	-	-	-	-	0.87	0.31	-	-	1.18
*r*_1_ = 2.0										
A-series	0.84	1.57	15.37	17.86	6.88	2.17	0.47	0.08	-	45.25
B-series	-	-	1.73	17.14	20.70	7.42	1.99	0.54	-	49.60
C-series	-	-	-	-	0.92	2.86	0.92	0.31	0.14	5.15

**Table 4:** Hydrolysis of the silanes in Figure 6 in acidic and alkaline media. Reprinted with permission from [46]. Copyright Springer Nature, 2003.

Compound	Hydrolysis Rate at pH 4 h^−1^	Hydrolysis Rate at pH 9 h^−1^
**1a**	21.8	24.0
**1b**	14.4	67.9
**1c**	^a^	17.5
**2a**	^b^	1.2
**2b**	27.2	9.5
**2c**	^a^	2.2
**3a**	1.6	1.2
**3b**	^c^	0.5
**4a**	31.5	27.7
**4b**	4.9	1.6
**5**	5.2	0.6
**6**	13.7	6.4

^a^ Hydrolysis of compounds **1c** and **2c**, respectively, was extremely fast; after 60 seconds, only the silanol could be observed besides liberated methanol; ^b^ The rate constant for the first hydrolysis step of compound **2a** could not be determined due to excessive overlap of the signals under the chosen conditions; ^c^ Hydrolysis of compound **3b** was found to be extremely slow under acidic conditions; almost no reaction was found to proceed within 1 h.

**Table 5:** The chemical shifts from the Q-species (hydrolyzed monomers and oligomers) of TEOS and TMOS. Here, the Q represents the quaternary functional silanes. Reprinted with permission from [4]. Copyright Elsevier, 1990.

		**Q^0^**	**Si(OR)*_x_*(OH)_4−*x*_**		
			**x**		
	4	3	2	1	0
TMOS	−78.5	−77.0	−75.5	−74.2	−73.1
TEOS	−81.95	−79.07	−76.58	−74.31	-
		**Q^0^**	**Q^1^**	**Q^2^**	**Q^3^**
TMOS					
–OCH_3_ substituted					
Monomer Q^0^		−78.47	-		
Dimer (Q^1^)_2_		-	−85.81		
Trimer Q^1^Q^2^Q^1^			−85.99	−93.69	
Cyclic trimer (Q^2^)_3_				−83.3 (possibly hydrolyzed)	
Cyclic tetramer (Q^2^)_4_				−92.9	
Linear tetramer Q^1^Q^2^Q^2^Q^1^			−85.98	−93.9	
Branched tetramer Q^1^Q^3^Q^1^Q^1^			−86.19		−102.10
TEOS					
–OC_2_H_5_ substituted					
Monomer Q^0^		−81.95			
Dimer (Q^1^)_2_			−88.85		
Trimer Q^1^Q^2^Q^1^			−88.99	−96.22	

**Table 6:** Assignment of the FTIR peaks for silanes.

Peak (cm^−1^)	Assignments
1198 or 1085	Si-O-CH_3_ [2,28]
1097	Si-O-Si (for the body) [2,19,24,28,59]
1032	Si-O-Si (for the linear) [2,24,28,49,59]
851	Si-O-Fe [2]
1167, 957	Si-O-C (rocking) [49,87]
940, 913	Si-OH [49,57,87]
877, 859, 2195	Si-H [57]
1104	Si-O-C [57]
830	Silica network [57]
1095, 1067	(T^1^)_2_(OH)_4_ ladder structure [24]
996	Open cage (T^3^)_4_((T^2^)_2_(OH)_2_ [24]
1135	T_8_ cage like [24]
1051–1057	Strained structure (T_6_) [59]
1120–1130	Unstrained structures (T_8_ and T_10_) [59]

**Table 7:**
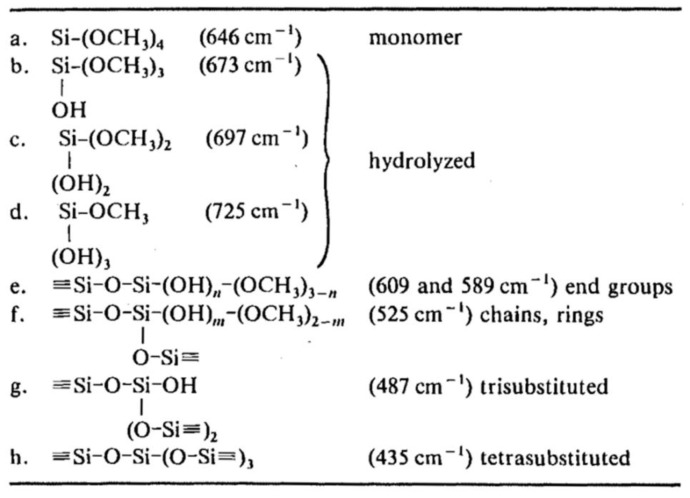
The Raman peaks of the TMOS species. Reprinted with permission from [4]. Copyright Elsevier, 1990.

**Table 8:** The assignment of vibrations for Raman peaks of trifunctional silanes. Data from [54]. Copyright John Wiley & Sons, 2016.

Raman Bands (cm^−1^)	Assignments
308, 350	O-Si-O bending
Between 250 and 500	Si-O-Si bending
430, 526	Si-O-C bending
622	Si-C stretching
647, 741	Si-O breathing
805	Si-O stretching
810	T_4_ (silsesquioxanes)
1089	Si-O-C stretching
840	Si-O-Si stretching
710	Hydrolyzed silane

## Supplementary Materials

The following are available online at https://www.mdpi.com/2073-4360/11/3/537/s1, Table S1: Commonly used silanes.
