# Supplementary material for: Kinetics of Alkoxysilanes and Organoalkoxysilanes Polymerization: A Review
Source: Polymers (Basel). 2019 Mar 21;11(3):537. doi: 10.3390/polym11030537 (PMC6473841; doi:10.3390/polym11030537)
Supplement: Supplementary file 1 [file polymers-11-00537-s001.pdf]

Supplementary Materials: Kinetics of Alkoxysilanes and Organoalkoxysilanes Polymerization: A Review

Ahmed A. Issa and Adriaan S. Luyt

Table S1. Commonly used silanes.

**Silane**	**Silane**
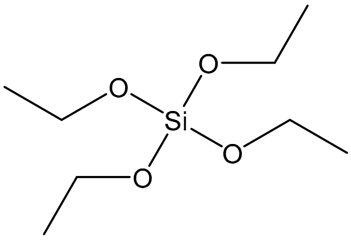<br><br>**Abbreviation:** TEOS<br>**Name:** Tetraethoxy silane OR tetraethyl orthosilicate<br>**Molecular weight:** 208.3 g mol^{-1}<br>**Density:** 0.933 g cm^{-3}	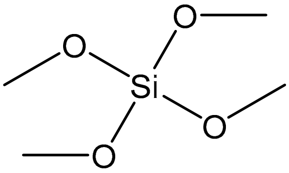<br><br>**Abbreviation:** TMOS<br>**Name:** Tetramethoxy silane, tetramethyl orthosilicate<br>**Molecular weight:** 152.3 g mol^{-1}<br>**Density:** 1.023 g cm^{-3}
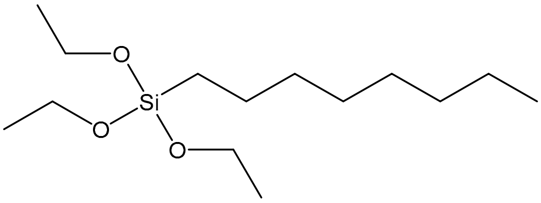<br><br>**Abbreviation:** OTES or OES<br>**Name:** Octyl triethoxysilane OR triethoxy(octyl)silane<br>**Molecular weight:** 276.2 g mol^{-1}<br>**Density:** 0.88 g cm^{-3}	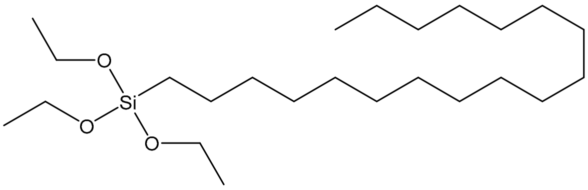<br><br>**Abbreviation:** ODTES<br>**Name:** Octadecyl triethoxysilane OR triethoxy(octadecyl)silane<br>**Molecular weight:** 416.8 g mol^{-1}<br>**Density:** 0.883 g cm^{-3}
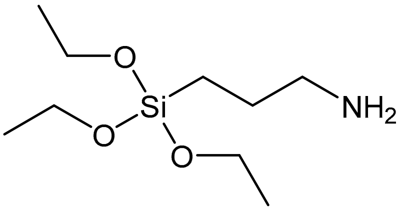<br><br>**Abbreviation:** APTS, PES, or γ-APS<br>**Name:** (3-Aminopropyl)triethoxysilane<br>**Molecular weight:** 221.4 g mol^{-1}<br>**Density:** 0.946 g cm^{-3}	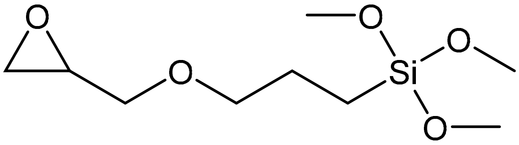<br><br>**Abbreviation:-** GPS, γ-GPS, or GTMS<br>**Name:** (3-Glycidyloxypropyl)trimethoxysilane<br>**Molecular weight:** 236.3 g mol^{-1}<br>**Density:** 1.07 g cm^{-3}

<table>
<tr>
<td>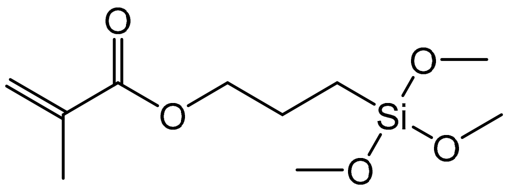
Abbreviation: MPMS, γ-MPS, and MPTMS
Name: 3-(Trimethoxysilyl)propyl methacrylate
Molecular weight: 248.4 g mol^{-1}
Density: 1.045 g cm^{-3}</td>
<td>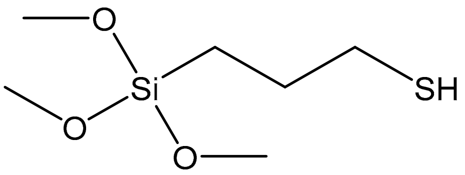
Abbreviation: MRPMS or McPTMS
Name: 3-mercaptopropyl trimethoxy silane
Molecular weight: 196.4 g mol^{-1}
Density: 1.057 g cm^{-3}</td>
</tr>
<tr>
<td>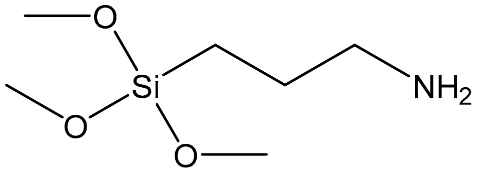
Abbreviation: PTMS
Name: 3-aminopropyl trimethoxy silane
Molecular weight: 179.3 g mol^{-1}
Density: 1.027 g cm^{-3}</td>
<td>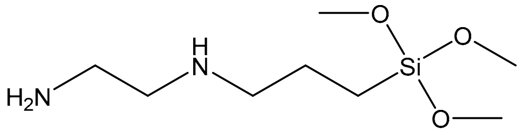
Abbreviation: DAMS
Name: 3-(2-aminoethylamino)propyl trimethoxy silane
Molecular weight: 222.4 g mol^{-1}
Density: 1.028 g cm^{-3}</td>
</tr>
<tr>
<td colspan="2">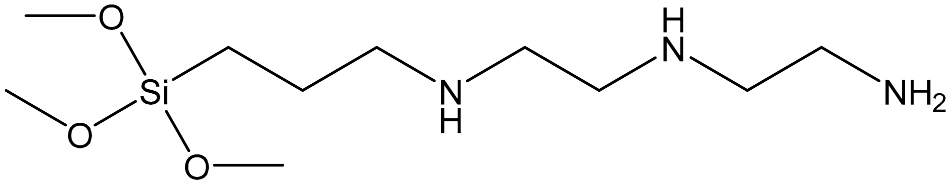
Abbreviation: TAMS
Name: 3-[2-(2-aminoethylamino) ethylamino]propyl trimethoxy silane
Molecular weight: 265.4 g mol^{-1}
Density: 1.03 g cm^{-3}</td>
</tr>
<tr>
<td>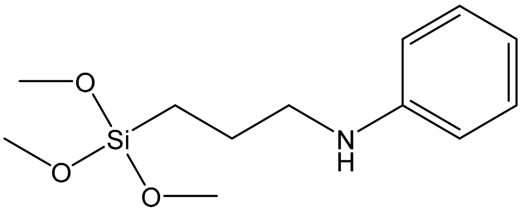
Abbreviation: PAPMS
Name: trimethoxy [3-(phenylamino)propyl] silane
Molecular weight: 225.4 g mol^{-1}
Density: 1.07 g cm^{-3}</td>
<td>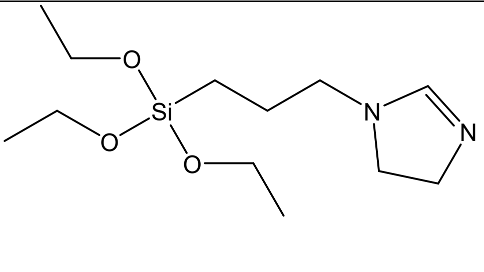
Abbreviation: IZPES
Name: triethoxy-3-(2- imidazolin-1-yl) propyl silane
Molecular weight: 274.4 g mol^{-1}
Density: 1.005 g cm^{-3}</td>
</tr>
</table>

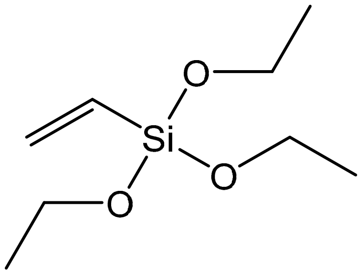<br>**Abbreviation:** VES<br>**Name:** vinyl triethoxy silane<br>**Molecular weight:** 190.3 g mol^{-1}<br>**Density:** 0.903 g cm^{-3}	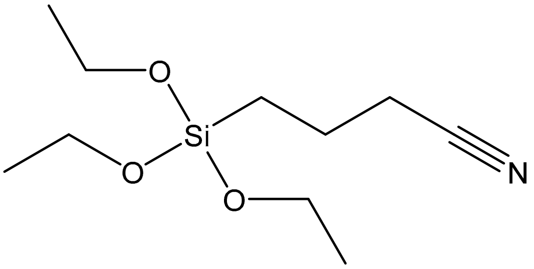<br>**Abbreviation:** Cyano, CPES, or 4-TBN<br>**Name:** 3-cyanopropyl triethoxy silane<br>**Molecular weight:** 231.4 g mol^{-1}<br>**Density:** 0.966 g cm^{-3}
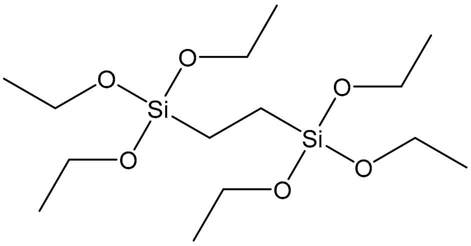<br>**Abbreviation:** BTSE<br>**Name:** bis-1,2-(triethoxysilyl)ethane<br>**Molecular weight:** 354.6 g mol^{-1}<br>**Density:** 0.958 g cm^{-3}	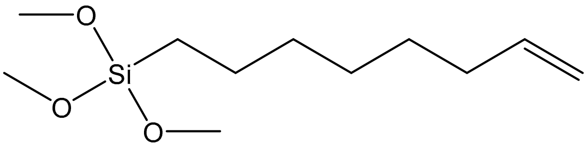<br>**Abbreviation:** OEMS<br>**Name:** (7-octen-1-yl) trimethoxy silane<br>**Molecular weight:** 232.4 g mol^{-1}<br>**Density:** 0.928 g cm^{-3}
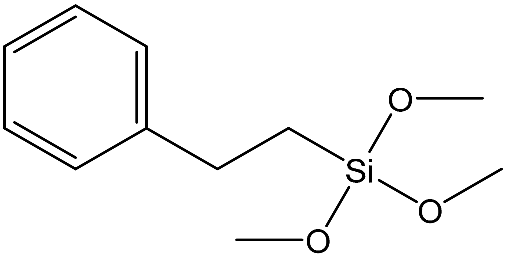<br>**Abbreviation:** PEMS<br>**Name:** trimethoxy (2-phenylethyl) silane<br>**Molecular weight:** 226.4 g mol^{-1}<br>**Density:** 1.033 g cm^{-3}	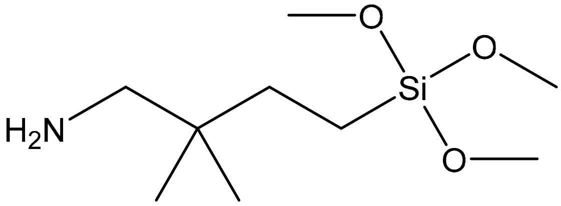<br>**Abbreviation:** ADBMS<br>**Name:** 4-amino-3,3-dimethyl butyl trimethoxy silane<br>**Molecular weight:** 221.4 g mol^{-1}<br>**Density:** -

<table>
<tr>
<td>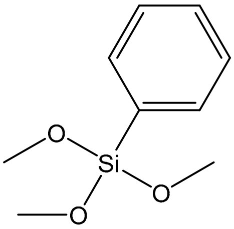
Abbreviation: PTMS
Name: Phenyl trimethoxy silane
Molecular weight: 198.3 g mol^{-1}
Density: 1.062 g cm^{-3}</td>
<td>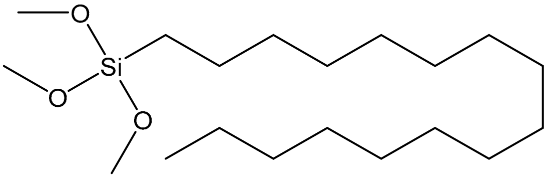
Abbreviation: HDTMS
Name: Hexadecyl-trimethoxy-silane
Molecular weight: 346.6 g mol^{-1}
Density: 0.89 g cm^{-3}</td>
</tr>
<tr>
<td>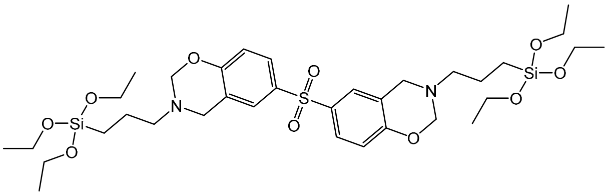
Abbreviation: BS-b
Name: triethoxysilane-based benzoxazine
Molecular weight: 741.1 g mol^{-1}
Density: -</td>
<td>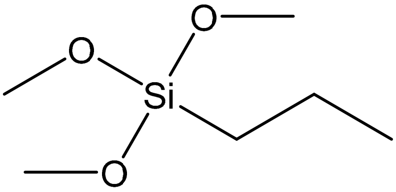
Abbreviation: nPrTMS
Name: propyl trimethoxy silane
Molecular weight: 164.3 g mol^{-1}
Density: 0.932 g cm^{-3}</td>
</tr>
<tr>
<td colspan="2">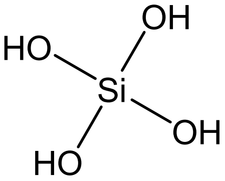
Abbreviation: silicic acid
Name: silicic acid
Molecular weight: 96.1 g mol^{-1}
Density: -</td>
</tr>
<tr>
<td>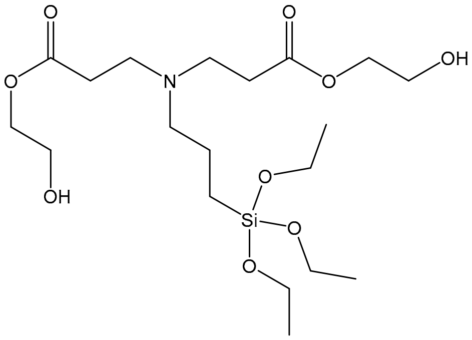
Abbreviation: -
Name: bis(2-hydroxyethyl) 3,3′-((3-(triethoxysilyl)propyl)azanediyl)dipropionate
Molecular weight: 453.6 g mol^{-1}
Density: -</td>
<td>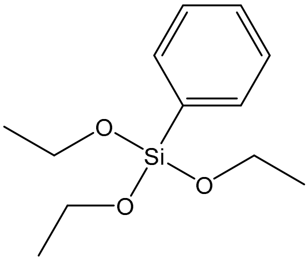
Abbreviation: PhTES or PTES
Name: Phenyltriethoxysilane
Molecular weight: 240.4 g mol^{-1}
Density: 0.996 g cm^{-3}</td>
</tr>
</table>

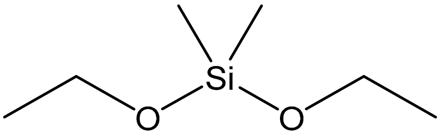<br><br>**Abbreviation:** DMDEOS<br>**Name:** dimethyldiethoxysilane<br>**Molecular weight:** 148.3 g mol^{-1}<br>**Density:** 0.865 g cm^{-3}	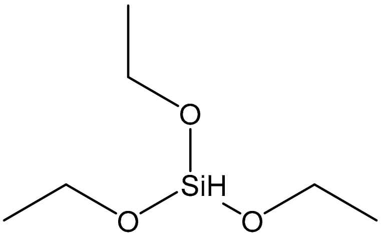<br><br>**Abbreviation:** TES<br>**Name:** Triethoxysilane<br>**Molecular weight:** 164.3 g mol^{-1}<br>**Density:** 0.89 g cm^{-3}
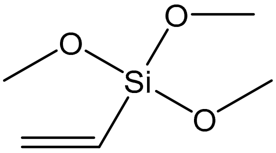<br><br>**Abbreviation:** VMS<br>**Name:** Vinyl trimethoxy silane<br>**Molecular weight:** 148.2 g mol^{-1}<br>**Density:** 0.968 g cm^{-3}	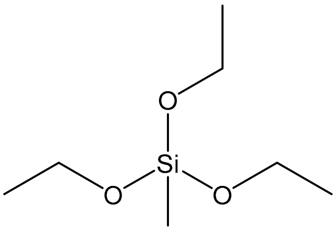<br><br>**Abbreviation:** MTES<br>**Name:** Methyltriethoxysilane<br>**Molecular weight:** 178.3 g mol^{-1}<br>**Density** 0.895 g cm^{-3}
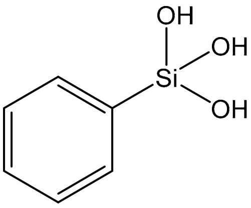<br><br>**Abbreviation:** phenylsilanetriol<br>**Name:** phenylsilanetriol<br>**Molecular weight:** 156.2 g mol^{-1}<br>**Density:** -	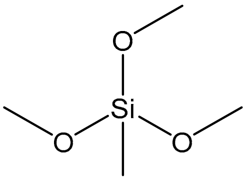<br><br>**Abbreviation:** MTMS<br>**Name:** Methyltrimethoxy silane<br>**Molecular weight:** 136.2 g mol^{-1}<br>**Density:** 0.955 g cm^{-3}
